# Hydroxytyrosol, the Major Phenolic Compound of Olive Oil, as an Acute Therapeutic Strategy after Ischemic Stroke

**DOI:** 10.3390/nu11102430

**Published:** 2019-10-11

**Authors:** Jesús Calahorra, Justin Shenk, Vera H. Wielenga, Vivienne Verweij, Bram Geenen, Pieter J. Dederen, Mᵃ Ángeles Peinado Herreros, Eva Siles, Maximilian Wiesmann, Amanda J. Kiliaan

**Affiliations:** 1 Department of Experimental Biology, University of Jaén, Campus Las Lagunillas s/n, 23071 Jaén, Spain; jgmoreno@ujaen.es (J.C.); apeinado@ujaen.es (M.Á.P.H.); esiles@ujaen.es (E.S.); 2 Radboud University Medical Center, Donders Institute for Brain, Cognition & Behaviour, Radboud Alzheimer Center, Department of Anatomy, Preclinical Imaging Centre PRIME, 6500 HB Nijmegen, The Netherlands; Justin.Shenk@radboudumc.nl (J.S.); Vera.Wielenga@radboudumc.nl (V.H.W.); Vivienne.Verweij@radboudumc.nl (V.V.); Bram.Geenen@radboudumc.nl (B.G.); Jos.Dederen@radboudumc.nl (P.J.D.); Maximilian.Wiesmann@radboudumc.nl (M.W.)

**Keywords:** stroke, hydroxytyrosol, dietary treatment, neuroinflammation, cerebral connectivity, cerebral blood flow, MRI, animal model

## Abstract

Stroke is one of the leading causes of adult disability worldwide. After ischemic stroke, damaged tissue surrounding the irreversibly damaged core of the infarct, the penumbra, is still salvageable and is therefore a target for acute therapeutic strategies. The Mediterranean diet (MD) has been shown to lower stroke risk. MD is characterized by increased intake of extra-virgin olive oil, of which hydroxytyrosol (HT) is the foremost phenolic component. This study investigates the effect of an HT-enriched diet directly after stroke on regaining motor and cognitive functioning, MRI parameters, neuroinflammation, and neurogenesis. Stroke mice on an HT diet showed increased strength in the forepaws, as well as improved short-term recognition memory probably due to improvement in functional connectivity (FC). Moreover, mice on an HT diet showed increased cerebral blood flow (CBF) and also heightened expression of brain derived neurotrophic factor (Bdnf), indicating a novel neurogenic potential of HT. This result was additionally accompanied by an enhanced transcription of the postsynaptic marker postsynaptic density protein 95 (Psd-95) and by a decreased ionized calcium-binding adapter molecule 1 (IBA-1) level indicative of lower neuroinflammation. These results suggest that an HT-enriched diet could serve as a beneficial therapeutic approach to attenuate ischemic stroke-associated damage.

## 1. Introduction

Stroke is a leading cause of death and long-term disability worldwide. Whereas two-third of stroke deaths occur in less developed countries, it is the second most common cause of death in Europe [1]. Ischemic stroke, caused by obstruction of a blood vessel by, for example, a thrombus is the most common type of stroke (80–85%). The neuronal injury after ischemic stroke is caused by the absence of oxygen and glucose during the ischemic period and, more importantly, by oxidative stress and increase in inflammation along the reperfusion period [2]. Consequently, neurodegeneration, especially in the core of the infarct, takes place, giving rise to a gradual and continuous deterioration of behavioral and cognitive functions [3,4]. The penumbra, the ischemic boundary zone around the irreversibly injured core, is a potentially salvageable tissue and may be the objective of restorative interventions [5].

Endovascular interventions and intravenous thrombolysis restore brain perfusion and limit the acute effects of stroke. However, no further stroke treatments are available, except for some rehabilitative therapies such as training, progressive task-related practice of skills, and neurostimulation [6]. The decrease of the oxidative stress level, the reduction of the inflammatory processes, or the stimulation of neuro- and synaptogenesis are some of the strategies that, particularly when combined, could help to reduce the impact of stroke in the potentially salvageable tissue. A dietary approach could play an essential role in this field. In fact, several studies prove its significance [7]. Our group has already demonstrated that a multi-nutrient intervention (Fortasyn) containing long-chain polyunsaturated fatty acids (LCPUFAS), extra vitamins, and antioxidants improves sensorimotor integration, brain integrity, and neurogenesis after ischemic stroke induction [8,9]. The PREDIMED trial, which studies the effect of Mediterranean diets in health, has highlighted the positive association between extra-virgin olive oil (EVOO) consumption and the risk of stroke in humans [10,11]. This beneficial effect of EVOO has also been shown to be protective in terms of redox homeostatic balance, lipid and protein damage, activation of NO synthase (NOS) in the penumbra, and reduction of apoptosis levels in chronic ischemic models [12,13,14].

EVOO, obtained by mechanical processes under cold temperatures, consists of two fractions: the major saponificable fraction (98%), composed of fatty acids such as oleic acid, and the minor unsaponificable fraction (2%), containing more than 230 components, amongst which are the phenolic alcohols [15]. Hydroxytyrosol (HT), together with tyrosol and oleuropein, are the most abundant phenolic alcohols in EVOO [16]. A number of studies have demonstrated that many of the beneficial properties of EVOO are strongly associated with HT. This polyphenol has shown numerous biological effects such as antioxidant and anti-inflammatory capacity, antitumor properties, and neuroprotective effects [17]. Until now, all studies on the neuroprotective effect of HT under ischemic conditions have been carried out ex vivo, using thick brain sections [18,19,20,21]. These sections were incubated with different concentrations of HT or extracted from previously treated animals. The results obtained from these experiments indicate that HT exerts a neuroprotective effect associated with lower release of lactic dehydrogenase, decreased levels of nitrosative and oxidative stress, and a decrease in inflammation. The neuroprotective effect of this compound in ischemic processes has also been studied in diabetic rats and indicated that its neuroprotective action is not exclusively linked to its antioxidant action [22].

With this background, the objective of the present study is to longitudinally evaluate the effect of a HT-enriched diet both on motor and cognitive skills as well as structural and functional MRI outcomes like cerebral blood flow (CBF) and grey and white matter integrity at day 7 and day 35 post-stroke in a well-known and broadly used mouse transient middle cerebral artery occlusion (tMCAo) model. In addition, metabolic, neurogenic, and inflammatory markers will be evaluated, as well as oxidative levels in serum, in order to investigate the potential of HT as an acute therapeutic strategy after stroke.

## 2. Materials and Methods

### 2.1. Animals

The present study was double-blinded randomized and performed at the Preclinical Imaging Center (PRIME) of the Radboud university medical center (Radboudumc, Nijmegen, The Netherlands) using 28 male 2–3 months old C57BL/6JRj mice (Harlan Laboratories Inc., Horst, The Netherlands). Before tMCAO, the mice had ad libitum access to standard food pellets (Ssniff rm/h V1534-000, Bio Services, Uden, The Netherlands) and autoclaved water and were individually housed in DVC cages (Digital Ventilated Cage, Tecniplast S.P.A., Buguggiate (VA), Italy) during the experiments to study individual locomotion via calculation of DVC metric measures (activity, walked distance, walked velocity, total turns, laterality index) during day and night time, before and after surgery [23,24]. The animals were kept on an artificial 12 h light-dark cycle (lights on at 7:00) in rooms controlled for humidity and temperature (21 ± 1 °C) and background music playing during the light cycle. All experiments were performed in accordance with the Dutch federal law for animal experimentation (“Wet op de Dierproeven,” 1996) and the regulations of the European Union Directive of 22 September 2010 (2010/63/EU). All experiments were approved by the Animal Ethics Committee of the RadboudUMC (protocol number: RU-DEC 2017-0021) and performed according to the ARRIVE guidelines.

### 2.2. Transient Middle Cerebral Artery Occlusion (tMCAo)

**Figure 1:**
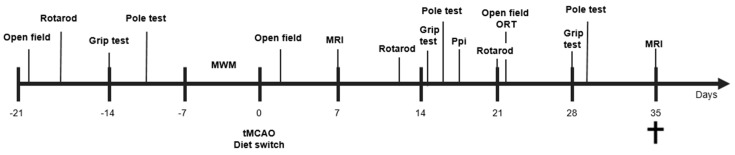
Study design. After a transient occlusion of the middle cerebral artery (tMCAo) for 30 min, mice were divided into two dietary groups (Control or HT-enriched). At seven and 35 days post-tMCAo, all mice underwent Magnetic Resonance Imaging (MRI). In between, all mice were tested on motor and cognitive impairments via several behavioral tests, like the Open field, Rotarod, Pole test, Prepulse inhibition (Ppi), grip strength test, and novel object recognition test (ORT). After MRI at day 35, animals were sacrificed, serum samples were recollected, and all brains were processed for immunohistochemical stainings and qPCR analysis.

At ~3 months of age, mice underwent transient (30 min) occlusion of the right middle cerebral artery (tMCAo), as previously described [9]. Mice were anesthetized with 1.5% isoflurane in a 2:1 (air:oxygen) mixture and were kept under anaesthesia for the duration of the surgery. Just prior to the occlusion procedure, a Laser Doppler probe (moorVMS-LDF2, Moor Instruments, Axminster, Devon, UK) was placed on the skull of the mice to monitor cerebral blood flow (CBF) as an assessment of the efficacy of the occlusion (≥ 80% loss of CBF). A 7-0 monofilament (190–200 μm, coating length 2–3 mm, 70SPRePK5, Doccol Corp., Sharon, MA, USA) was inserted in the right carotid cerebral artery (CCA) and pushed upward to the proximal part of the middle cerebral artery (MCA). The filament occluded the MCA for 30 min, after which it was retracted to allow reperfusion. As a control, part of the mice underwent sham surgery instead of tMCAo. In these mice, the filament was immediately retracted after touching the Willis’ circle. After surgery, all mice were carefully assessed for pain and other discomfort, weighed every day at 12:00 for seven days, and food intake was monitored. Exclusion criteria were decreased motor activity (<50% of the baseline measurements combined from the baseline values of each behavioral test) or extreme weight loss (>20% within three consecutive days). Using a T2-weighted RARE sequence to measure lesion size and ratio between stroke (right) and unaffected (left) hemispheres, all stroke animals showed a comparable lesion size at seven days post-stroke and no dietary effect on lesion size (data not shown). Notably, both dietary groups demonstrated atrophy over time in a decrease in left-to-right ratio (D7: 0.97 ± 0.03; D35: 0.85 ± 0.08; F(1,12) = 31.3, *p* < 0.006). The time line of the experimental design is illustrated in Figure 1.

### 2.3. Group Allocation and Diet

After stroke, mice were randomly allocated, using a random sequence generator, to one of two diets: an HT-enriched diet (*n* = 13; stroke (*n* = 6), sham (*n* = 7)) or an isocaloric control diet (*n* = 15; stroke (*n* = 8), sham (*n* = 7)). Group sizes were calculated according to effect sizes (*p* = 0.05, statistical power: 0.80), exclusion, and mortality rates previously determined by a similar study of our group [9]. Both diets contained 24.0% kcal protein, 15.0% kcal fat, and 61.0% kcal carbohydrates (Research Diet Services B.V., Wijk bij Duurstede, The Netherlands), and their detailed composition is described elsewhere [25,26,27]. The HT-enriched diet was supplied with 0.03% HT (Seprox Biotech, Murcia, Spain) incorporated into the pellets, resulting in approximately 45 mg HT/kg bw/day (human equivalent dose of 3.6 mg HT/kg bw/day). Food intake and body weight was measured before and during the weeks after surgery. Excluded mice per test are shown in Table S1.

### 2.4. Open Field

Mice were placed in a square open field (45 × 45 × 30 cm) for 10 min to assess locomotion and explorative behavior. The open field test was performed three times: once prior to surgery, and at three and 21 days post-surgery. Locomotion was automatically recorded, using EthoVision XT 10.1 (Noldus, Wageningen, The Netherlands). In EthoVision, the floor of the open field arena was divided in zones to distinguish the periphery, corners, and center. The frequency of entering these zones was automatically recorded. Additionally, manual scoring of exploratory behaviors (sitting, walking, grooming, wall-leaning, rearing) was performed, as previously described [28].

### 2.5. Grip Test

Grip strength of the mice was measured with a grip strength meter (Grip Strength Meter, 47200, Ugo Basile, Italy) at three time points: pre-stroke and day 15 and day 29 (post-stroke). Mice were held by their tail so they could grab a trapeze or grid (connected to the grip strength meter) to measure, respectively, muscle strength in the fore limbs (trapeze) or strength in all four limbs collectively (grid). Trials in which mice grabbed the trapeze with only one forepaw or the grid with less than four paws were excluded. Additionally, trials in which the mouse grabbed the side of the trapeze were also excluded. The maximum value of peak force (in gram per force (gf)) was averaged per experimental group for both trapeze and grid.

### 2.6. Pole Test

The pole test is used to monitor motor function. It was performed pre-stroke and post-stroke at day 14 and day 28. The mouse was placed on a vertical pole with its head pointed upward and had to turn 180 degrees to walk down the pole. The time needed to fully turn 180 degrees (turning time) and the turning direction were manually recorded. Additionally, video recordings of each trial were automatically analyzed with EthoVision XT 10.1 (Noldus, Wageningen, The Netherlands) to calculate the velocity (cm/s) with which the mouse walked down the pole. Trials that were excluded from statistical analysis consisted of every first trial of a mouse (acclimatization), trials in which the mouse was already turning when placed on the pole, and trials in which the mouse showed no motivation and was assisted by the researcher to go down.

### 2.7. Rotarod

As a measure of balance, coordination, physical condition and motor planning the Rotarod (ITC LifeScience Inc., Woodland Hills, CA, USA) was performed pre-surgery and at day 10 and day 21 post-surgery. The mice were placed on the Rotarod and left to acclimatize for 10–30 s. Then, the Rotarod was turned on to accelerate for 300 s from 4 to 40 rpm. The latency time to fall was recorded. No significant effects (data not shown) were found.

### 2.8. Prepulse Inhibition (Ppi)

The Prepulse inhibition (Ppi) test was performed 16 days post-stroke to examine the sensorimotor gating integration of the mice, as previously described [29]. To measure the startle reactivity, the mouse was placed in a restrainer in the chamber of the SR-LAB startle response system (San Diego Instruments, San Diego, CA, USA) and exposed to blocks of startle pulses. Pre-pulse inhibition was calculated during the second block of startle pulses as 100 - response to startle pulse after pre-pulse/response to startle pulse × 100%. Additionally, habituation to startle pulses was investigated by comparing the startle response to the first startle pulse block to the startle response in the third (last) startle pulse block.

### 2.9. Morris Water Maze (MWM)

The Morris water maze (MWM) was used to test spatial learning and memory in rodents. In short, before surgery, all mice were placed in a circular pool, filled with opaque water, and were trained to find a submerged platform in the northeast (NE) quadrant of the pool by using distant visual cues. At the end of the fourth day, mice additionally performed a single probe trial, in which the platform was removed from the swimming pool. Mice were allowed to swim for 120 s, and the time spent swimming and searching in the NE quadrant (where the platform had been located) was recorded. The MWM is used to analyze spatial learning and memory before surgery (data not shown). All mice learned to find the hidden platform revealed by a decrease in latency time from acquisition day 1 to day 4 (−16.33s ± 5.68s; F (3,81) = 3.8, *p* < 0.013).

### 2.10. Novel Object Recognition Test (ORT)

Short-term memory of the mice was measured with the novel object recognition test (ORT), as previously described [9]. This test spanned three days. On the first day, mice were acclimatized to the open field box by letting them explore freely for 10min. On the second and third day, mice underwent object recognition trials. First, mice performed a familiarization trial. In this trial two identical objects (eggs, tea light holders, yellow plastic ice cream cones, or bottles filled with sand) (F1 and F2) were placed in the open field, equidistant from the center, and the mouse was then placed in the open field to freely explore the objects for 4 min. After a certain delay (30 min on day 2 and 60 min on day 3), the trial was repeated, with one of the familiar objects (F3) and one object replaced for a novel object (N1). Exploratory behavior of the mice was measured using EthoVision XT 10.1 (Noldus, Wageningen, The Netherlands) as direct contact with the object, or movement within a 2cm diameter around the object.

To measure object recognition, several indexes were calculated in both the familiarization and the test phase. In the test phase, discrimination between objects was calculated with the discrimination index (DI), as the time spend around N1 minus F3, divided by the time spend around both objects (DI = (N1 − F3)/(N1 + F3)). From this index, a number between +1 and −1 is obtained, where closer to +1 shows more time spend around N1, closer to −1 more time spend around F3, and 0 shows no difference in time spend at either object. To measure recognition memory, the recognition index (RI) was calculated as the time spend around N1 as a fraction of the time spend around both objects (RI = N1/ (N1 + F3)). The preference for either object was calculated with the preference index (PI), as time the mouse spend around N1 (or F3) as a percentage of the time spend around both objects (PI = 100 × ([N1 or F3]/(N1 + F3)). If N1 is the numerator, closer to 100% indicates preference for N1, 50% indicates no preference, and below 50% preference for F3 (vice versa if F3 is the numerator) [30].

### 2.11. Digital Ventilated Cages (DVC)

As previously described [23,24], animals were single housed in DVC cages during the experiment to study individual locomotion via calculation of DVC metric measures (activity, walked distance, walked velocity, total turns, laterality index) during day and night time before and after surgery. A detailed explanation of the calculations on the aforementioned DVC metric measures can be found in the Supplementary Materials (Figure S1). For analysis of the processed results, we compared week 1–5 individually to pre-surgery values.

### 2.12. In Vivo Magnetic Resonance Imaging (MRI)

MRI measurements were performed seven and 35 days after surgery with an 11.7T BioSpec Avance III small animal MR system (Bruker BioSpin, Ettlingen, Germany) operating on Paravision 6.0.1. software (Bruker, Karlsruhe, Germany) under full isoflurane anaesthesia (3.5% for induction and 1.8% for maintenance; in a 2:1 (medical air:oxygen) mixture). Body temperature was monitored with a rectal probe, and maintained at 37 °C using hot air flow. A pneumatic cushion respiratory monitoring system (Small Animal Instruments Inc., Stony Brook, New York, NY, USA) was used to measure the respiration rate of the mouse. Mice with scans that showed motion and/or echo planar imaging artifacts were excluded from MRI analysis.

### 2.13. Arterial Spin Labelling (ASL)

To assess cerebral blood flow (CBF), perfusion imaging was performed using a flow-sensitive alternating inversion recovery arterial (FAIR) technique as previously described [5,9]. Hippocampus, cerebral cortex, and thalamus, according to the Paxinos and Franklin atlas [31], were analyzed as regions of interest (ROI) by a researcher blinded to the surgery and treatment groups. For each ROI, CBF was analyzed in the affected (ipsilateral/right) and unaffected (contralateral/left) hemisphere separately for each group.

### 2.14. Diffusion Tensor Imaging (DTI)

Diffusion of water was imaged as described previously [32,33,34]. In short, 22 axial slices covering the whole brain were acquired with a four-shot SE-EPI protocol. B0 shift compensation, navigator echoes, and an automatic correction algorithm to limit the occurrence of ghosts and artefacts were implemented. Encoding b-factors of 0 s/mm^2^ (b0 images; 5×) and 1000 s/mm^2^ were used, and diffusion-sensitizing gradients were applied along 30 non-collinear directions in three-dimensional space. The diffusion tensor was estimated for every voxel using the PATCH algorithm [35]. Mean water diffusivity (MD) and fractional anisotropy (FA) were derived from the tensor estimation following a protocol as described elsewhere [34]. MD and FA values were measured in several white matter (WM) and grey matter (GM) areas, manually selected based on an anatomical atlas [36].

### 2.15. Resting State Functional MRI (rs-fMRI)

Subsequently after the acquisition of the anatomical reference images, resting state functional Magnetic Resonance Imaging (rsfMRI) datasets were acquired using a single-shot spin-echo sequence with echo-planar readout (SE-EPI) sequence. Six hundred repetitions with a repetition time (TR) of 1.8 s and echo time of 16.9 ms were recorded for a total acquisition time of 18 min. The rsfMRI datasets were first realigned using a least-squares method and rigid-body transformation with Statistical Parametric Mapping (SPM) mouse toolbox (SPM5, University College London; http://www.fil.ion.ucl.ac.uk/spm/; Sawiak et al., 2009). Mean and maximum displacement across the six degrees of freedom (along the x-, y-, and z-axes and on three rotation parameters pitch, roll, and yaw) were measured in each mouse. The mean SE-EPI images for each mouse were then used to generate a study-specific template through linear affine and nonlinear diffeomorphic transformation (ANTs. v1.9; http://picsl.upenn.edu/ANTS/). Visual inspection of the normalized dataset was performed to screen for possible normalization biases. On the template, 12 areas were selected in left and right hemisphere. The selected regions were based on previous work concerning functional connectivity in mice [37] and included left and right dorsal hippocampus, left and right ventral hippocampus, left and right auditory cortex, left and right motor cortex, left and right somatosensory cortex, and left and right visual cortex. All cortical ROIs were selected 1–2 voxels away from the edge of the cortex to minimize the impact of susceptibility artefacts, which are more prominent in areas close to tissue interfaces (e.g., near the skull or near the ear canals). In-plane spatial smoothing (0.4 × 0.4 mm), linear detrending, and temporal high-pass filtering (cut-off at 0.01 Hz) were applied to compensate for small across-mouse misregistration and temporal low-frequency noise. Functional connectivity (FC) group comparison between ROIs were calculated from the BOLD time series using total correlation analyses implemented in FSLNets (FSLNets v0.3; www.fmrib.ox.ac.uk/fsl). Pearson’s correlation values were Fisher transformed to Z-scores for group comparisons and statistical analysis.

### 2.16. qPCR

RNA was isolated from frontal parts (divided in to left and right) of the brain (bregma: −0.10 to 4.28 using TRIzol method (Thermo Scientific, Waltham, MA, USA). The samples were treated with RNase-free DNase I (RQ1, Promega, Fitchburg, MA, USA) to eliminate any genomic DNA. cDNA was synthesized using the iScript kit (Bio-Rad, Hercules, CA, USA). qPCR was done in 96-well plates (Thermo Scientific) using a StepOnePlus system (Thermo Scientific). Gene expression of postsynaptic density protein 95 (Psd-95), brain derived neurotrophic factor (Bdnf), and glucose transporter 1 (GLUT-1) were quantitatively assessed by using hypoxanthine guanine phosphoribosyl transferase (Hprt) and beta-2 microglobulin (B2m) as the normalizing genes. The sequences of primers are shown in Table S2.

### 2.17. (Immuno)histochemistry

After the last scanning session, the mice were sacrificed by transcardial perfusion using 0.1 M phosphate-buffered saline (PBS) followed by 4% paraformaldehyde in 0.1 M PBS. The brains were harvested and stored separately. The brains were postfixed overnight in 4% paraformaldehyde at 4 °C and transferred to 0.1M PBS containing 0.01% sodium azide the next day. One part of the brain (bregma: −0.1 to −4.36) was cut in 30 μm frontal sections using a sliding microtome (Microm HC 440, Walldorf, Germany) equipped with an object table for freeze-sectioning at −60 °C. Then, 24 h before cutting, the brains were transferred to 30% sucrose in 0.1 M phosphate buffer. Eight series were cut and stored in 0.1 M PBS with 0.01% sodium azide so multiple immunohistochemical stainings could be performed.

All sections were stained in one session to minimize differences in staining intensity. In total three stainings were performed for vascular integrity measured via glucose transporter-1 (GLUT-1), for activated microglia via ionized calcium-binding adapter molecule 1 (IBA-1) as indicator for neuroinflammation, and for immature neurons (measure for neurogenesis) with antibodies against doublecortin (DCX) on free-floating brain sections on shaker tables at room temperature. Immunohistochemistry was performed using standard free-floating labelling procedures, using previously described protocols [38]. The GLUT-1 amount was visualized using polyclonal rabbit anti-GLUT-1 antibody (1:40,000, Chemicon AB 1340, Chemicon International, Inc., Temecula, CA, USA) and as secondary antibody donkey anti-rabbit biotin (1:1500 Jackson ImmunoResearch, West Grove, PA, USA). For IBA-1, as primary antibody against IBA-1 polyclonal goat anti-IBA-1 (1:3000; Abcam, Cambridge, UK) and for DCX, polyclonal goat anti-DCX (1:8000; Santa Cruz Biotechnology Inc., Santa Cruz, CA, USA) was used as a primary antibody to assess neurogenesis. For both, donkey anti-goat biotin (1:1500; Jackson ImmunoResearch) was used as a secondary antibody. A more frontal part of the brain tissue (bregma: −0.10 to 0.98) was fixed in 4% paraformaldehyde in 0.1 M phosphate buffer (pH 7.4) and embedded in paraffin according to a standard protocol.

### 2.18. Quantification (GLUT-1, IBA-1, and DCX)

Brain sections (bregma: −1.46 to −2.30) were preselected for quantification accordingly to the atlas of Franklin and Paxinos [31]. Quantification was done on images taken with a 5× objective using an Axio Imager A2 (Zeiss Germany). ImageJ (National Institute of Health, Bethesda, MD, USA) was used to analyze the regions of interest (GLUT-1 + IBA-1: Cortex (bregma 0.62 & −1.94), hippocampus, thalamus, caudate putamen, and corpus callosum (only IBA-1); DCX: Hippocampus).

### 2.19. Determination of Serum NO Level

Nitric oxide production was indirectly quantified by determining nitrate/nitrite and S-nitroso compounds (NOx), using an ozone chemiluminescence-based assay adapted to serum samples [39,40]. In brief, serum samples were deproteinized with 0.8 N NaOH and 16% ZnSO4 solutions (1/0.5/0.5). After centrifugation at 10,000× *g* for 5 min, the resulting supernatants were removed for chemiluminescence analysis [41] in an NO analyzer (NOA 280i; Sievers Instruments, Boulder, CO, USA). NOx concentration was calculated by comparison with standard solutions of sodium nitrate. Final NOx values were expressed as µM.

### 2.20. Determination of Serum Oxidative Stress Level

Thiobarbituric acid reactive substances (TBARS), a major indicator of oxidative stress, was determined using an adaptation of the method described by Buege and Aust [42]. Specifically, 8% sodium dodecyl sulfate was added (1:1) to each serum sample. Samples were vortexed and mixed (1:6) with a reagent containing 15% trichloroacetic acid, 0.38% thiobarbituric acid, and 2% HCl and then heated for 30 min at 96 °C, cooled, and centrifuged (3000× *g* for 5 min). The supernatants were collected, and the absorbance was measured at 532 nm. The concentration of TBARS was determined by extrapolation from a malondialdehyde standard curve. Results were expressed as µM.

### 2.21. Statistical Analyses

A random and blinded selection procedure was maintained throughout the study. Group means were compared using univariate analysis of variance (ANOVA) with Bonferroni correction for multiple testing with a statistical program, SPSS 24 (IBM SPSS Statistics 24, IBM Corporation, Armonk, NY, USA). Nonparametric tests were used when assumptions of normality and homogeneity of variance were not met. *p*-values lower than 0.05 were considered significant. Data are presented as mean ± SEM.

## 3. Results

### 3.1. Food Intake and Body Weight

**Figure 2:**
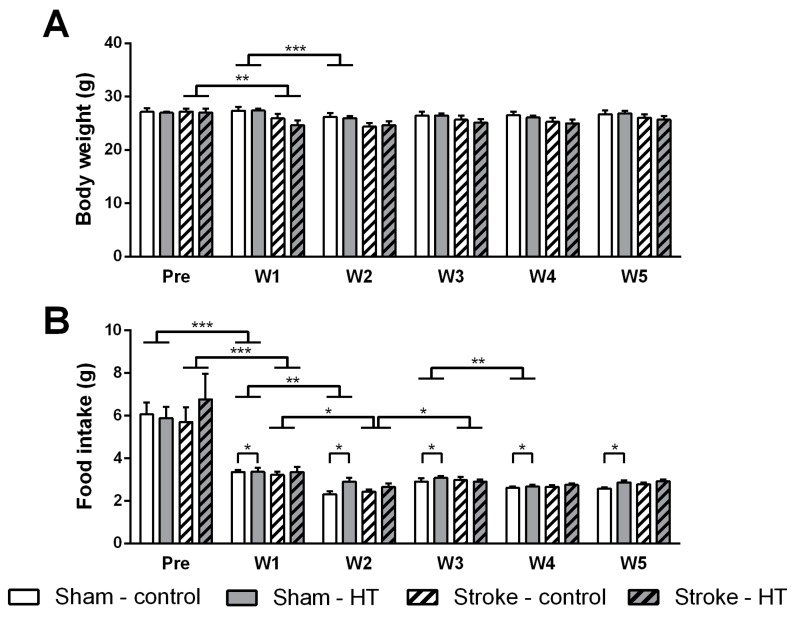
Effect of hydroxytyrosol (HT) diet on body weight and food intake. (**A**) Body weight of both dietary stroke groups decreased post-stroke versus pre-stroke (*p* < 0.002). Sham mice lost body weight comparing week 1 with week 2 (*p* < 0.001), with no effect in the upcoming weeks. (**B**) Food intake of both stroke and sham mice decreased over time comparing pre-surgery and the first week after surgery (Sham: *p* < 0.001; Stroke: *p* < 0.001). Sham mice had a lower food intake comparing week 1 to week 2 (*p* < 0.006) and also week 3 to week 4 (*p* < 0.009). Sham mice on HT diet had a higher food intake during all post-surgery weeks than sham mice on control diet (*p* < 0.041). Stroke mice ate less comparing week 1 with week 2 (*p* < 0.025) and started to eat more comparing week 2 with 3 (*p* < 0.008). Only sham mice showed a significant time interaction in body weight (*p* < 0.001) and in food intake (*p* < 0.001). Values represent mean ± SEM. * *p* < 0.05, ** *p* < 0.01, *** *p* < 0.001.

Food intake and body weight was measured before and during the weeks after surgery (Figure 2). Body weight (Figure 2A) of sham mice did not decrease over time comparing pre-surgery with the first week after surgery (F(1,12) = 3.3, *p* < 0.095), while body weight of both dietary stroke groups decreased post-stroke versus pre-stroke (F(1,12) = 18.2, *p* < 0.002). Food intake (Figure 2B) of both stroke and sham mice decreased over time comparing pre-surgery with the first week after surgery (Stroke: F(1,12) = 19.8, *p* < 0.001; Sham: F(1,12) = 40.5, *p* < 0.001).

Investigating the development of body weight and food intake over time, these parameters were analyzed weekly after surgery. Sham mice showed a significant time interaction in body weight (F(1,48) = 14.5, *p* < 0.001) and also in food intake (F(1,48) = 16.5, *p* < 0.001). In detail, sham mice lost body weight comparing week 1 with week 2 (*p* < 0.001), while this effect was not present in the upcoming weeks comparing week 2 with week 3, week 3 with week 4, and week 4 with week 5. Sham mice had a lowered food intake comparing week 1 to week 2 (*p* < 0.006) and also week 3 to week 4 (*p* < 0.009). Notably, sham mice on HT diet had a higher food intake during all post-surgery weeks than sham mice on control diet (F(1,12) = 5.3, *p* < 0.041). Stroke mice demonstrated a time interaction in food intake (F(1,48) = 10.2, *p* < 0.001). After further statistical analysis, stroke mice ate less comparing week 1 with week 2 (*p* < 0.025) and started to eat more comparing week 2 with 3 (*p* < 0.008).

### 3.2. Behaviour, Cognition, and Motor Tasks

#### 3.2.1. Open Field

**Figure 3:**
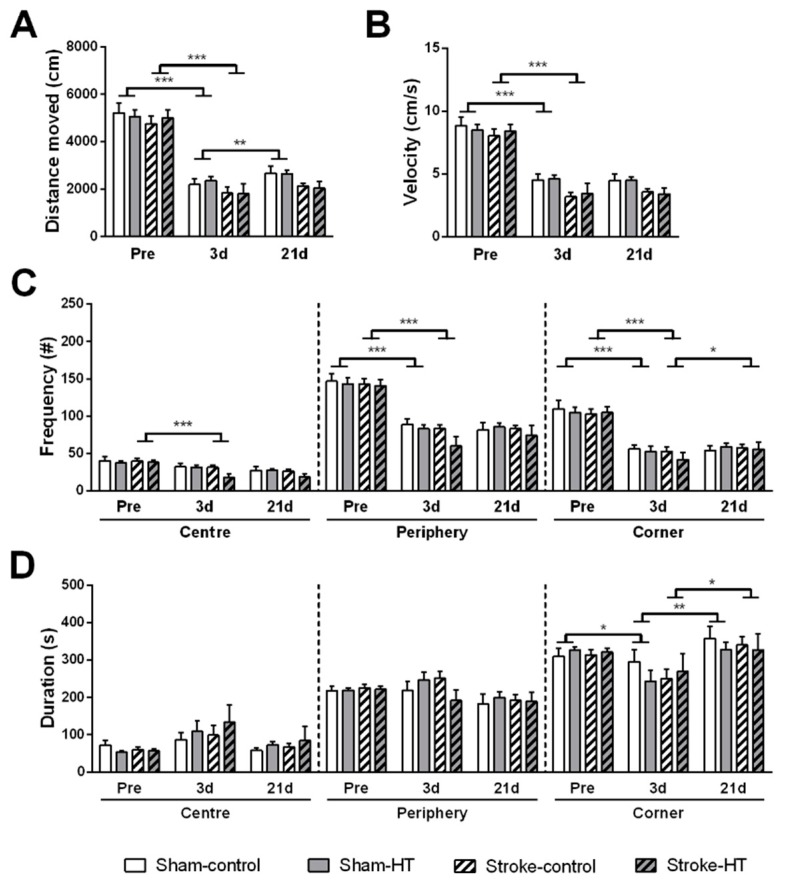
Activity, anxiety and explorative behavior measured in open field prior to stroke and at three and 21 days post-stroke. Locomotion was assessed by evaluating (**A**) the distance moved and (**B**) the velocity. Anxiety and exploration were evaluated by tracking (**C**) the frequency and (**D**) the time spent in the different zones in the arena (center, periphery and corner). (**A**,**B**) At three days post-surgery, all sham and all stroke mice moved a shorter distance (Sham: *p* < 0.001; Stroke: *p* < 0.001) with lower velocity (Sham: *p* < 0.001; Stroke: *p* < 0.001) compared to baseline. At 21 days post-stroke, sham animals walked more in the open field compared to three days post-stroke animals (*p* < 0.002). (**C**,**D**) Stroke mice visited the center (*p* < 0.001), periphery (*p* < 0.001) and corners (*p* < 0.001) less frequently at three days post-stroke. Sham animals also visited the periphery (*p* < 0.001) and corners (*p* < 0.001) less frequently at three days post-stroke compared to baseline, however they spent less time in the corners (*p*< 0.024) at three days. Values represent mean ± SEM.* *p* <0.05, ** *p* < 0.01, *** *p* < 0.001.

Mice were placed in open field to assess locomotion and explorative behavior (Figure 3). At three days post-surgery, all sham and all stroke mice moved a shorter distance (Sham: F(1,12) = 137.1, *p* < 0.001; Stroke: F(1,12) = 116.2, *p* < 0.001) (Figure 3A) and with a lower velocity (Sham: F(1,12) = 99.4, *p* < 0.001; Stroke: F(1,12) = 116.5, *p* < 0.001) in the arena (Figure 3B), compared to baseline. At 21 days post-surgery, sham animals walked more in the open field compared to three days post-surgery (F(1,12) = 17.1, *p* < 0.002), but stroke animals did not (Figure 3A). No diet effects were observed in distance moved and velocity in the open field.

Anxiety and exploration were assessed by tracking the position of the mice in the open field (center, corners, periphery). Compared to baseline, stroke mice visited the center (F(1,12) = 60.7, *p* < 0.001), periphery (F(1,12) = 230.2, *p* < 0.001), and corners (F(1,12) = 85.5, *p* < 0.001) less frequently at three days post-stroke (Figure 3C) but showed no change in time spent at all locations (Figure 3D). Sham animals also visited the periphery (F(1,12) = 73.4, *p* < 0.001) and corners (F(1,12) = 172.0, *p* < 0.001) less frequently at three days post-stroke compared to baseline (Figure 3C), but they spent less time in the corners (F(1,12) = 6.7, *p* < 0.024) at three days (Figure 3D). No diets effects were observed in these parameters.

**Figure 4:**
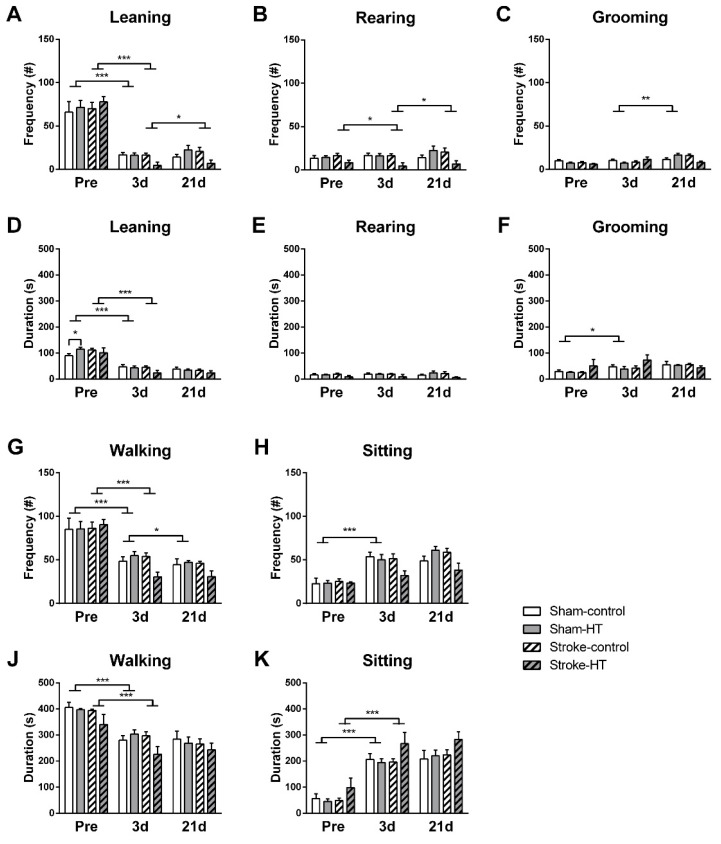
Behaviors in the open field. The behaviors of the mice in the arena during the open field were manually scored as another measure of locomotion, activity, and explorative behavior. (**A**–**C**,**G**,**H**) Frequency and (**D**–**F**,**J**,**K**) duration of leaning, rearing, grooming, sitting, and walking were quantified pre-surgery and three and 21 days after surgery. (**A**,**G**) All mice showed decreased frequency of wall leaning (Sham: *p* < 0.001; Stroke: *p* < 0.001) and walking (Sham: *p* < 0.001; Stroke: *p* < 0.001) and (**D**,**J**) also spent less time performing these behaviors (Wall leaning: Sham: *p* < 0.001; Stroke: *p* < 0.001; Walking: Sham: *p* < 0.001; Stroke: *p* < 0.001) at three days post-stroke, (**K**,**F**) while they spent more time sitting (Sham: *p* < 0.001) and grooming (Sham: *p* < 0.028). (**C**) At baseline and three days post-stroke, sham-HT mice groomed less frequently than sham-control mice (*p* < 0.036; not shown in graph). (**B**) Stroke mice also reared less frequently at three days compared to pre-stroke (*p* < 0.012). (**G**) At 21 days post-stroke, walking frequency (*p* < 0.026) was even further decreased in sham mice, (**A**,**B**) whereas stroke show increased rearing frequency (*p* < 0.012) and wall leaning frequency (*p* < 0.020). (**C**) Sham mice groomed more frequently at 21 days compared to three days post-stroke (*p* < 0.004). Values represent mean ± SEM.* *p* < 0.05, ** *p* < 0.01, *** *p* < 0.001.

The frequency with which the mice engaged in different types of exploratory behavior was manually scored (Figure 4). Compared to baseline, all mice showed decreased frequency of wall leaning (Sham: F(1,12) = 64.5, *p* < 0.001; Stroke: F(1,12) = 258.1, *p* < 0.001) and walking (Sham: F(1,12) = 26.7, *p* < 0.001; Stroke: F(1,12) = 157.3, *p* < 0.001) and also spent less time performing these behaviors (*Wall leaning*: Sham: F(1,12) = 123.0, *p* < 0.001; Stroke: F(1,12) = 78.1, *p* < 0.001; Walking: Sham: F(1,12) = 84.2, *p* < 0.001; Stroke: F(1,12) = 40.3, *p* < 0.001) at three days post-stroke, while they spent more time sitting (Sham: F(1,12) = 172.4, *p* < 0.001; Stroke: F(1,12) = 62.3, *p* < 0.001) and grooming (Sham: F(1,12) = 6.2, *p* < 0.028). At baseline and three days post-stroke, sham-HT mice groomed less frequently than sham-control mice (F(1,12) = 5.6, *p* < 0.036; not shown in graph). Additionally, stroke mice also reared less frequently at three days compared to pre-stroke (F (1,12) = 8.6, *p* < 0.012). At 21 days post-stroke, walking frequency (F (1,12) = 6.4, *p* < 0.026) was even further decreased in sham mice, whereas stroke mice showed no change in walking frequency or duration but did show increased rearing (F(1,12) = 8.6, *p* < 0.012) and wall leaning frequency (F(1,12) = 7.2, *p* < 0.020). Sham mice groomed more frequently at 21 days compared to three days post-stroke (F (1,12) = 12.3, *p* < 0.004). No additional diet effects were found for these parameters (Figure 4).

#### 3.2.2. Grip Test

**Figure 5:**
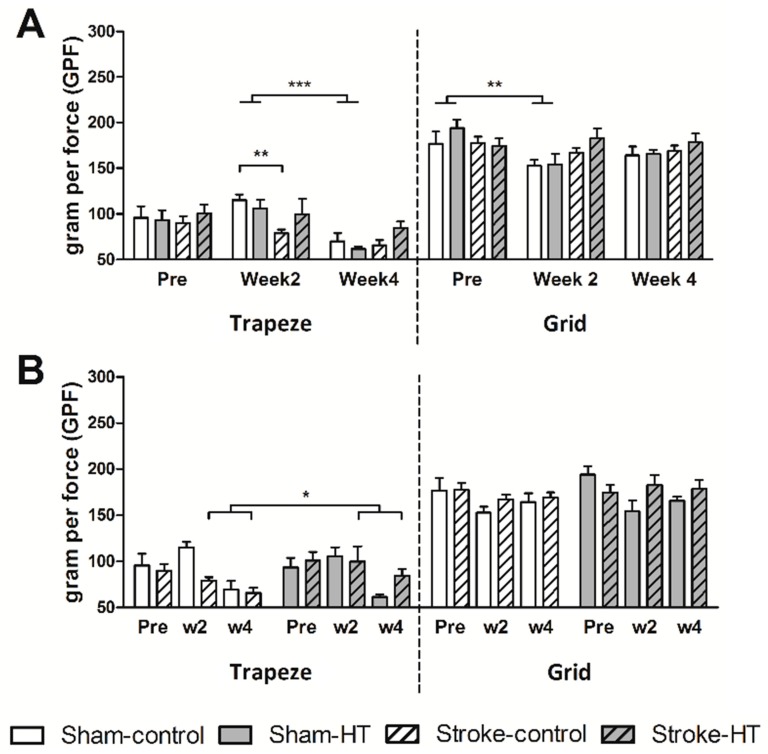
Grip strength in the forelimbs (trapeze) and in all four paws (grid) before (pre) and after (weeks 2 and 4) stroke. (**A**) Time effects and (**B**) diet effects on maximum grip strength are shown. (**A**) At week 2, sham mice showed a lower grip strength on the grid compared to pre-surgery (*p* < 0.004). A surgery effect was observed at week 2, shown by weaker grip strength in forelimbs in stroke-control mice compared to sham-control mice (*p* < 0.002). At four weeks post-surgery, sham mice showed a decreased forelimb grip strength compared to two weeks post-surgery (*p* < 0.001). (**B**) HT-fed stroke mice demonstrated a higher grip strength on the trapeze at weeks 2 and 4 compared to stroke control diet-mice (*p* < 0.018). Values represent mean ± SEM. * *p* < 0.05, ** *p* < 0.01, *** *p* < 0.001.

Forelimb strength of the mice was quantified with a grip test by letting the mice grip a small trapeze connected to a grip strength meter (Figure 5). Fore- and hind-limb strength was determined in a similar fashion, using a grid instead of a trapeze. At week 2, only sham mice showed a lower grip strength on the grid compared to pre-surgery (F (1,12) = 13.0, *p* < 0.004) (Figure 5A). Stroke mice showed no significant decrease in grip strength on either the trapeze or grid 2 weeks after stroke compared to baseline. A surgery effect was observed at week 2, shown by weaker grip strength in forelimbs in stroke-control mice compared to sham-control mice (F (1,9) = 17.3, *p* < 0.002) (Figure 5A). At four weeks post-surgery, sham mice show a decreased forelimb grip strength compared to two weeks post-surgery (F(1,10) = 26.0, *p* < 0.001) (Figure 5A). Interestingly, HT-fed stroke mice demonstrated a higher grip strength on the trapeze at weeks 2 and 4 compared to stroke control–diet mice (F (1,12) = 497.0, *p* < 0.018). No diet effects were observed on the grid (Figure 5B).

#### 3.2.3. Pole Test

**Figure 6:**
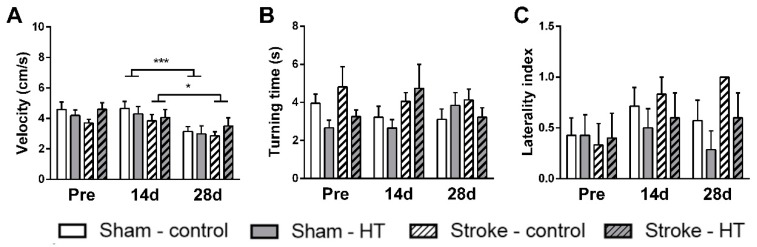
Motor coordination assessed pre-stroke and 14 days and 28 days post-stroke by the pole test. (**A**) Downwards walking velocity. (**B**) Time to turn around on the pole (turning time) and (**C**) tendency to turn right vs left (laterally index). (**A**) At 28 days post-surgery, sham mice (*p* < 0.001) and stroke mice (*p* < 0.049) walked down the pole with a lower velocity compared to 14 days post-surgery. Values represent mean ± SEM. Values represent mean ± SEM. * *p* < 0.05, *** *p* < 0.001.

The pole test was performed to assess motor dysfunction. The time needed to make a full 180 degree turn on the pole and the turning direction was manually scored, and the velocity with which the mice walked down the pole was calculated. There was no change in velocity between baseline measurement and the first measurement after surgery (at 14 days). At 28 days post-surgery, sham mice (F (1,12) = 21.4, *p* < 0.001) and stroke mice (F(1,9) = 5.2, *p* < 0.049) walked down the pole with a lower velocity compared to 14 days post-surgery (Figure 6). Neither time nor diet effects on turning side preference or turning time were observed.

#### 3.2.4. Prepulse Inhibition (Ppi)

**Figure 7:**
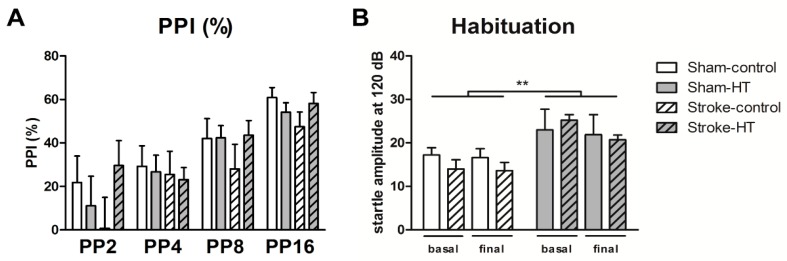
Sensorimotor integration measured before and after (16 days) stroke induction by the prepulse inhibition test (PPI). (**A**) PPI data shown as percentage. (**B**) Habituation to startle pulse. HT mice showed a higher startle amplitude to the basal and final startle stimulus of 120 dB than control mice (*p* < 0.009). Values represent mean ± SEM. ** *p* < 0.01.

The pre-pulse inhibition test was performed to assess sensorimotor gating after stroke. In both surgery and diet groups, no effects could be detected on PPI. No habituation effects were observed, but an overall diet effect was detected. HT mice showed a higher startle amplitude to the basal and final startle stimulus of 120 dB than control mice (F (1,22) = 8.3, *p* < 0.009) (Figure 7).

#### 3.2.5. Novel Object Recognition Test (ORT)

**Figure 8:**
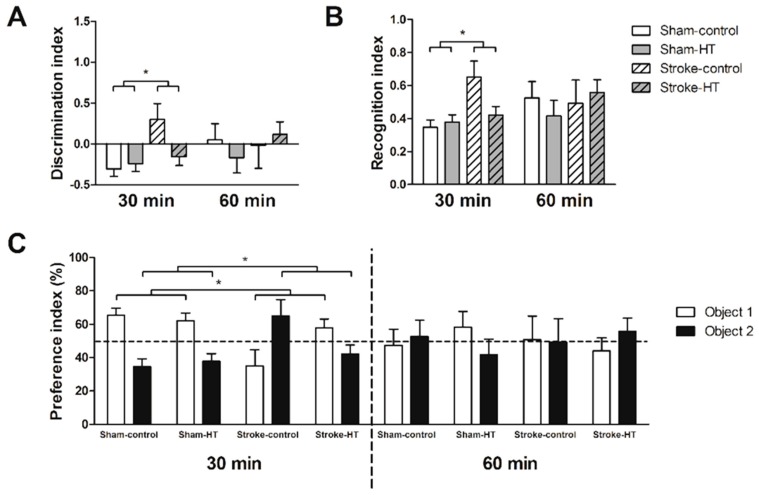
Familiarization phase object recognition test (ORT). Short-term memory of sham and stroke mice assessed by the novel ORT. (**A**) Discrimination index, (**B**) Recognition index, and (**C**) preference index. (**A**–**C**) Stroke animals had a preference for object 2 and sham animals a preference for object 1 (*p* < 0.026). Values represent mean ± SEM. * *p* < 0.05.

**Figure 9:**
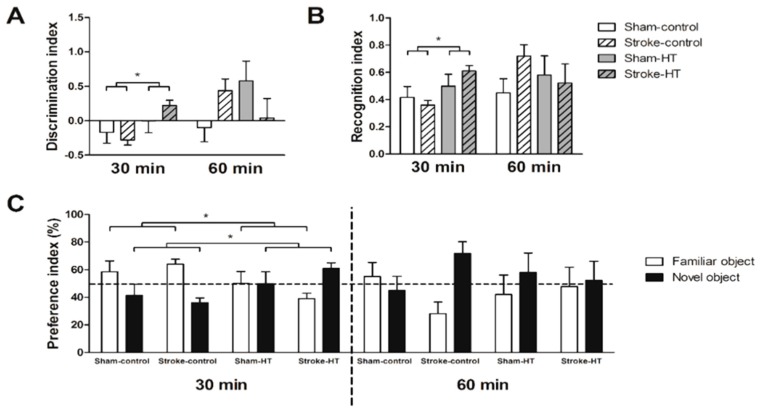
Test phase ORT. Short-term memory of sham and stroke mice assessed by the novel ORT. (**A**) Discrimination index, (**B**) Recognition index, and (**C**) preference index. (**A**–**C**) HT-fed animals showed a preference for the novel object and visited it more frequently than control diet-animals (*p* < 0.019). Values represent mean ± SEM. * *p* < 0.05.

The object recognition task (ORT) was performed once after stroke to measure short-term memory of the mice. In the 30 min trials of the familiarization phase, all discrimination index (DI), recognition index (RI), and preference index (PI) showed that stroke animals have a preference for object 2 and sham animals a preference for object 1, as they visited it more frequently (F(1,22) = 5.7, *p* < 0.026). However, in stroke animals, this effect was largely caused by mice on control diet, as they only showed as PI above 50% (Figure 8). In the 30 min trials of the test phase, HT-fed animals showed a preference for the novel object and visited it more frequently than control diet-animals (F(1,22) = 6.4, *p* < 0.019), as shown by all indexes (DI, RI, and PI) (Figure 9).

#### 3.2.6. Digital Ventilated Cages (DVC) Metrics

DVC were used to study individual locomotion via calculation of DVC metric measures (activity, walked distance, walked velocity, total turns, laterality index) during day and night time before and after surgery. No diet effects were found on DVC metrics.

##### Activity

Pre-surgery to Post-surgery Week 1

No effects of both types of surgery (stroke and sham) were found on daytime activity.

During the night time, only stroke mice were less active after surgery over time (F(1,12) = 5.3, *p* < 0.040).

Pre-surgery to Post-surgery Week 2

While sham mice were more active (F(1,12) = 6.7, *p* < 0.024) during daytime, stroke mice were more active (F(1,12) = 6.3, *p* < 0.028) during the night time.

Pre-surgery to Post-surgery Week 3

While during the day time both sham and stroke mice were more active (Sham: F(1,12) = 11.5, *p* < 0.005; Stroke: F(1,12) = 5.3, *p* < 0.040), during the night time, stroke mice were more active (F(1,12) = 11.4, *p* < 0.006).

Pre-surgery to Post-surgery Week 4

While during the night time stroke mice were more active (F(1,12) = 5.3, *p* < 0.040), during the day time, sham mice were more active (F(1,12) = 5.7, *p* < 0.035).

Pre-surgery to Post-surgery Week 5

While during the night time both sham and stroke mice were more active (Sham: F(1,12) = 5.1, *p* < 0.044; Stroke: F(1,12) = 9.4, *p* < 0.010), during the day time, sham mice were more active (F(1,12) = 6.7, *p* < 0.024).

##### Walked Distance

Pre-surgery to Post-surgery Week 1

No effects of both types of surgery (stroke and sham) nor diet effects were found on daytime walked distance.

During the night time, only sham mice walked less after surgery (F(1,12) = 12.0, *p* < 0.005).

Pre-surgery to Post-surgery Week 2

During both day and night time, sham mice walked less comparing pre-surgery with post-surgery week 2 (Daytime: F(1,12) = 9.8, *p <* 0.009; Nighttime: F(1,12) = 24.0, *p* < 0.001).

Pre-surgery to Post-surgery Week 3

During the day time, sham mice walked less comparing pre-surgery with post-surgery week 3 (F(1,12) = 10.4, *p* < 0.007).

Pre-surgery to Post-surgery Week 4

Only during the day time, both sham and stroke mice walked less comparing pre-surgery with post-surgery week 4 (Sham: F(1,12) = 16.9, *p* < 0.001; Stroke: F(1,12) = 7.3, *p* < 0.019).

Pre-surgery to Post-surgery Week 5

While during the day time all sham mice walked less (F(1,12) = 12.5, *p* < 0.004), during the night time, all stroke mice walked more comparing pre-surgery with post-surgery week 5 (F(1,12) = 9.6, *p* < 0.009).

##### Walked Velocity

Pre-surgery to Post-surgery Week 1

During both day and night time, both stroke (Daytime: F(1,12) = 36.4, *p* < 0.001; Nighttime: F(1,12) = 21.0, *p* < 0.001) and sham (Daytime: F(1,12) = 8.1, *p* < 0.015; Nighttime: F(112) = 37.0, *p* < 0.001) mice walked slower after surgery.

Pre-surgery to Post-surgery Weeks 2–5

No effects were found.

##### Turns

Pre-surgery to Post-surgery Week 1

During both day and night time, only sham (Daytime: F(1,12) = 7.4, *p* < 0.019; Nighttime: F(1,12) = 42.5, *p* < 0.001) mice turned less often after surgery.

Pre-surgery to Post-surgery Week 2

During both day and night time, only sham (Daytime: F(1,12) = 12.3, *p* < 0.004; Nighttime: F(1,12) = 9.5, *p* < 0.010) mice turned less often after surgery.

Pre-surgery to Post-surgery Week 3

No effects were found.

Pre-surgery to Post-surgery Week 4

During the night time, only stroke mice turned more often after surgery (F(1,12) = 7.8, *p* < 0.016).

Pre-surgery to Post-surgery Week 5

While during the day time only stroke mice turned more often (F(1,12) = 7.8, *p* < 0.016), during the night time, both sham and stroke mice turned more often (Sham: F(1,12) = 5.2, *p* < 0.041; Stroke: F(1,12) = 17.5, *p* < 0.001).

##### Laterality

Pre-surgery to Post-surgery Week 1

No effects were found.

Pre-surgery to Post-surgery Week 2

Only during the night time, stroke mice (F(1,12) = 6.0, *p* < 0.031) showed a left turning preference (laterality).

Pre-surgery to Post-surgery Weeks 3–5

No effects were found.

### 3.3. In Vivo Magnetic Resonance Imaging (MRI)

#### 3.3.1. Cerebral Blood Flow (CBF)

**Figure 10:**
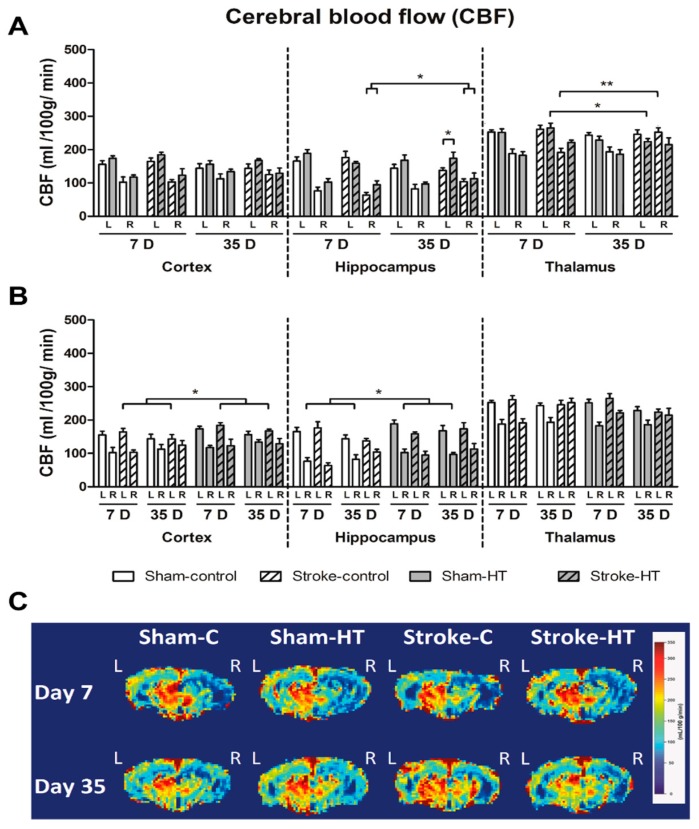
CBF was assessed in the lesioned and unlesioned hemisphere at seven and 35 days post-stroke in the hippocampus, thalamus, and cortex. At seven days post-stroke, cerebral blood flow (CBF) was lower in all groups in the right cortex (Sham-control: *p <* 0.002; Sham-HT: *p <* 0.004; Stroke-control: *p <* 0.007; Stroke-HT: *p <* 0.024), right hippocampus (Sham-control: *p <* 0.003; Sham-HT: *p <* 0.001; Stroke-control: *p <* 0.001; Stroke-HT: *p <* 0.016), and right thalamus (Sham-control: *p <* 0.002; Sham-HT: *p <* 0.001; Stroke-control: *p <* 0.004; Stroke-HT: *p <* 0.049) than in the corresponding regions of interest (ROI) in the left hemisphere (not shown in figure). At 35 days post-stroke, almost all groups still displayed a lower CBF in the right cortex (Sham-control: *p <* 0.10; Sham-HT: *p <* 0.017; Stroke-control: *p <* 0.012, Stroke-HT: *p <* 0.007) and right hippocampus (Sham-control: *p <* 0.004; Sham-HT: *p <* 0.001; Stroke-HT: *p <* 0.002) compared to the left cortex and left hippocampus, respectively (not shown in figure). In the right thalamus, a lower CBF was also observed in sham animals at 35 days, compared to the left thalamus (Sham-control: *p <* 0.002; Sham-HT: *p <* 0.001) (not shown in figure). CBF was also increased in the left cortex of stroke-HT mice compared to stroke-control mice at seven and 35 days post-stroke (**B**, *p <* 0.043). All stroke animals showed a significantly increased CBF at 35 days post-stroke in the right hippocampus and compared to seven days post-stroke (**A**, *p <* 0.015). Notably, CBF in the right hippocampus was increased in HT-fed sham mice of both surgery groups at both seven and 35 days post-surgery (**B**, Sham-HT: *p <* 0.046). In the left hippocampus, only HT-fed stroke mice maintained an increased CBF at 35 days compared to control diet-fed mice (*p <* 0.048). In the left thalamus, a decreased CBF was observed at 35 days compared to seven days post-stroke in stroke-HT mice (*p <* 0.036). Conversely, an increase of CBF was observed in the right thalamus in stroke-control mice at 35 days post-stroke, compared to seven days post-stroke (*p <* 0.003). (**C**) Representative high-resolution voxel-wise analyzed CBF images at seven and 35 post-stroke. Values represent mean ± SEM. Sham-control: n = 7, sham-HT: n = 6, stroke-control: n = 7, stroke-HT: n = 4. * *p <* 0.05, ** *p <* 0.01.

Using an ASL-FAIR technique, CBF was assessed in the lesioned and unlesioned hemisphere at seven and 35 days post-stroke in the hippocampus, thalamus, and cortex (Figure 10). At seven days post-stroke, CBF was lower in all groups in the right cortex (Sham-control: F(1,6) = 24.9, *p <* 0.002; Sham-HT: F(1,5) = 24.5, *p <* 0.004; Stroke-control: F(1,6) = 16.1, *p <* 0.007; Stroke-HT: F(1,4) = 12.6, *p <* 0.024), right hippocampus (Sham-control: F(1,6) = 24.2, *p <* 0.003; Sham-HT: F(1,5) = 50.5, *p <* 0.001; Stroke-control: F(1,6) = 70.1, *p <* 0.001; Stroke-HT: F(1,4) = 16.3, *p <* 0.016), and right thalamus (Sham-control: F(1,6) = 28.5, *p <* 0.002; Sham-HT: F(1,5) = 262.5, *p <* 0.001; Stroke-control: F(1,6) = 21.2, *p <* 0.004; Stroke-HT: F(1,3) = 10.3, *p <* 0.049) than in the corresponding ROI in the left hemisphere (not shown in figure).

At 35 days post-stroke, almost all groups still displayed a lower CBF in the right cortex (Sham-control: F(1,6) = 14.0, *p <* 0.10; Sham-HT: F(1,6) = 10.8, *p <* 0.017; Stroke-control: F(1,6) = 12.7, *p <* 0.012, Stroke-HT: F(1,5) = 18.9, *p <* 0.007) and right hippocampus (Sham-control: F(1,6) = 19.5, *p <* 0.004; Sham-HT: F(1,6) = 43.0, *p <* 0.001; Stroke-HT: F(1,5) = 33.5, *p <* 0.002) compared to the left cortex and left hippocampus, respectively. In the right thalamus, a lower CBF was also observed in sham animals at 35 days, compared to the left thalamus (Sham-control: F(1,6) = 25.2, *p <* 0.002; Sham-HT: F(1,6) = 38.8, *p <* 0.001). Interestingly, reperfusion was observed in stroke animals in the right thalamus, as demonstrated by the lack of significant difference in CBF between the left and right thalamus at 35 days (not shown in figure).

All stroke animals showed a significantly increased CBF at 35 days post-stroke in the right hippocampus and compared to seven days post-stroke (F(1,10) = 8.6, *p <* 0.015). In the left hippocampus, only HT-fed stroke mice maintained an increased CBF at 35 days compared to control diet-fed mice (F(1,10) = 5.1, *p <* 0.048). In the left thalamus, a decreased CBF was observed at 35 days compared to seven days post-stroke in stroke-HT mice (F(1,3) = 13.1, *p <* 0.036). Conversely, an increase of CBF was observed in the right thalamus in stroke-control mice at 35 days post-stroke, compared to seven days post-stroke (F(1,6) = 23.4, *p <* 0.003) (Figure 10A).

An overall diet effect was observed in the right hippocampus at seven and 35 days post-stroke, as shown by an increased CBF in HT-fed sham mice of both surgery groups (F(1,11) = 5.0, *p <* 0.046). Additionally, an increased CBF was also observed in the left cortex of stroke-HT mice compared to stroke-control mice at seven and 35 days post-stroke (F(1,10) = 5.4, *p <* 0.043) (Figure 10B).

#### 3.3.2. DTI and rs-fMRI

Fractional Anisotropy (FA)

**Figure 11:**
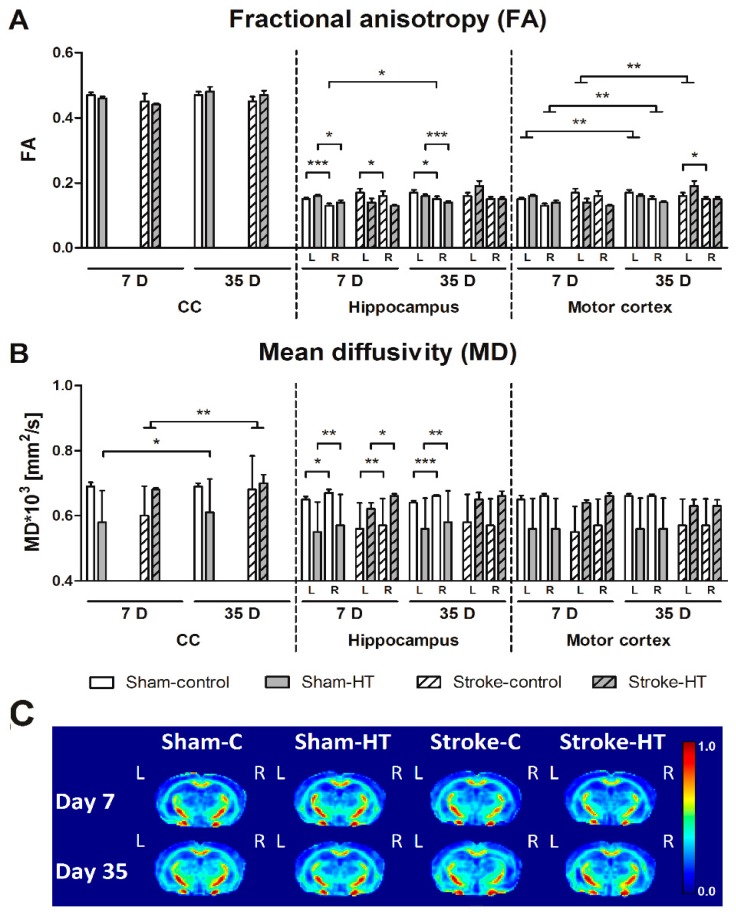
White matter (WM) and grey matter (GM) integrity as measured by quantitatively assessed diffusion tensor-derived indices at 7 + 35 days poststroke in mice fed HT or Control diet. Fractional anisotropy (FA) (**A**) and mean diffusivity (MD) (**B**) were measured for ROI drawn in white (Corpus Callosum, CC) and grey matter (Hippocampus, motor cortex) regions. (**A**) In sham-control (7D, *p <* 0.001; 35D, *p <* 0.011) and sham-HT mice (7D, *p <* 0.020; 35D, *p <* 0.001), FA in the right hippocampus was lower than in the left hippocampus at seven days and 35 days after surgery. Only in stroke-control mice at seven days post-stroke FA was decreased in the right hippocampus compared to its contralateral counterpart (*p <* 0.022). FA in the right hippocampus also increased between seven days and 35 days after surgery in sham-control mice (*p <* 0.035). A lowered FA in the right the motor cortex compared to left motor cortex was only found at 35 days in stroke-control (*p <* 0.033) mice. In the left motor cortex, stroke mice had an FA increase in the hemisphere at 35 days post-stroke compared to seven days post-stroke (*p <* 0.006). Sham animals showed a decrease of FA in both the left (*p <* 0.003) and right (*p <* 0.004) motor cortex over time (35 days vs. seven days). (**B**) MD was higher in the right hippocampus than in the left hippocampus at seven days post-surgery in all groups (Sham-control: *p <* 0.013; Sham-HT: *p <* 0.004; Stroke-control: *p <* 0.006; Stroke-HT: *p <* 0.050), while at 35 days, it was higher only in sham mice (Sham-control: *p <* 0.001; Sham-HT: *p <* 0.002). Only in stroke-control mice at seven days post-surgery, a higher MD in the right motor cortex than the left motor cortex was detected (*p <* 0.075). Sham-HT mice had a heightened MD of the corpus callosum at 35 days compared to seven days post-surgery (*p <* 0.033). All stroke mice also showed a higher MD in the corpus callosum 35 days compared to seven days post-surgery (*p <* 0.008). (**C**) Representative high-resolution set of FA images for each dietary group at seven and 35 days poststroke. * *p <* 0.05, ** *p <* 0.01, *** *p <* 0.001.

White matter (WM) and grey matter (GM) integrity as measured by quantitatively assessed diffusion tensor-derived indices at 7 + 35 days poststroke in mice fed HT or Control diet (Figure 11). In sham-control and sham-HT mice, the right hippocampus showed a lower FA than the left hippocampus at seven days (Sham-control: F(1,6) = 32.1, *p <* 0.001; Sham-HT: F(1,5) = 11.4, *p <* 0.020) and 35 days (Sham-control: F(1,6) = 13.1, *p <* 0.011; Sham-HT: F(1,5) = 49.7, *p <* 0.001) after surgery. In stroke mice, only the control group at seven days post-stroke showed that FA was lower in the right hippocampus than its contralateral counterpart (F(1,6) = 9.3, *p <* 0.022). In contrast, FA in the right hippocampus increased between seven days and 35 days after surgery in sham-control mice (F(1,6) = 7.4, *p <* 0.035). In the motor cortex, a decreased FA in the right compared to left hemisphere was only observed at 35 days in stroke-control (F(1,6) = 7.6, *p <* 0.033) mice and not in stroke-HT mice. Stroke mice showed an FA increase in the left motor cortex at 35 days post-stroke compared to seven days post-stroke (F(1,11) = 11.6, *p <* 0.006). However, sham animals showed a decrease of FA in both the left (F(1,11) = 13.7, *p <* 0.003) and right (F(1,11) = 12.8, *p <* 0.004) motor cortex over time (35 days vs. seven days) (Figure 11A).

Mean Diffusivity (MD)

In the hippocampus, MD was higher in the lesioned (right) hemisphere than in the unaffected (left) hemisphere at seven days post-surgery in all groups (Sham-control: F(1,6) = 12.0, *p <* 0.013; Sham-HT: F(1,5) = 25.8, *p <* 0.004; Stroke-control: F(1,6) = 17.4, *p <* 0.006; Stroke-HT: F(1,5) = 6.6, *p <* 0.050), and at 35 days only in sham mice (Sham-control: F(1,6) = 61.6, *p <* 0.001; Sham-HT: F(1,5) = 34.9, *p <* 0.002).

Sham-HT mice showed an increase in MD of the corpus callosum at 35 days compared to seven days post-surgery (F(1,5) = 8.5, *p <* 0.033). All stroke mice also showed a higher MD in the corpus callosum 35 days compared to seven days post-surgery (F(1,11) = 10.7, *p <* 0.008).

rsfMRI

Total Correlations

**Figure 12:**
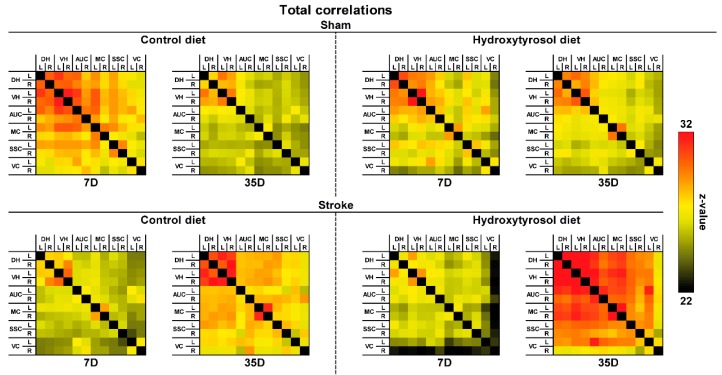
Resting-state functional connectivity (FC) based on total analyses in the brains of mice fed HT or Control diet seven and 35 days post-stroke. FC was measured between 12 ROI: dorsal hippocampus (DH), ventral hippocampus (VH), auditory cortex (AUC), motor cortex (MC), somatosensory cortex (SSC), and visual cortex (VC). Total correlations revealed that HT diet improved FC in stroke mice between several ROI, i.e., right DH to left MC (*p <* 0.022); right DH to right M1 (*p <* 0.040).

Only in stroke mice, HT diet improved FC from day seven to day 35 after stroke between several cerebral regions: right dorsal hippocampus (DHR) to left motor cortex (MCL, F(1,8) = 8.1, *p <* 0.022); DHR to right motor cortex (MCR, F(1,8) = 6.1, *p <* 0.040); MCL to right visual cortex (VCR, F(1,8) = 9.4, *p <* 0.014); VCR to MCR (F(1,8) = 5.5, *p <* 0.048) (Figure 12).

### 3.4. Immunohistochemistry and Biochemical Analyses

#### 3.4.1. DCX Staining

**Figure 13:**
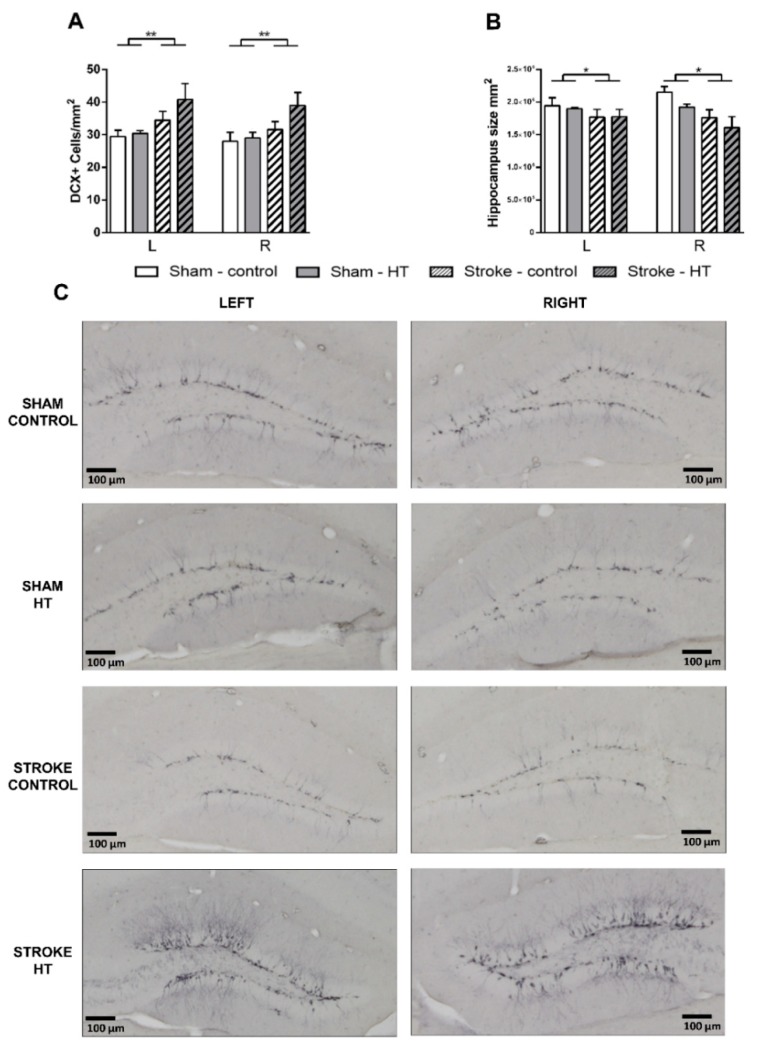
Immunohistochemical stainings for doublecortin (DCX) in hippocampus of the brains of HT and control fed mice 35 days after surgery. (**A**) All stroke mice showed an increased number of DCX+ cells/mm^2^ compared to sham mice (*p <* 0.008). (**B**) The hippocampus size was reduced significantly in stroke mice without a diet effect (*p <* 0.015). (**C**) Representative photos per experimental group are shown. Values represent mean ± SEM. * *p <* 0.05, ** *p <* 0.01.

To visualize immature neurons, we used an anti-DCX antibody as a neurogenesis marker. DCX+ cells were quantified in hippocampus (Figure 13C). Here, we found an increased number of DCX+ cells/mm^2^ in all stroke mice compared to sham mice (F(1,23) = 8.4, *p <* 0.008) (Figure 13A). The hippocampus size was reduced significantly in stroke mice without a diet effect (F(1,23) = 7.0, *p <* 0.015) (Figure 13B).

#### 3.4.2. IBA-1 Staining

**Figure 14:**
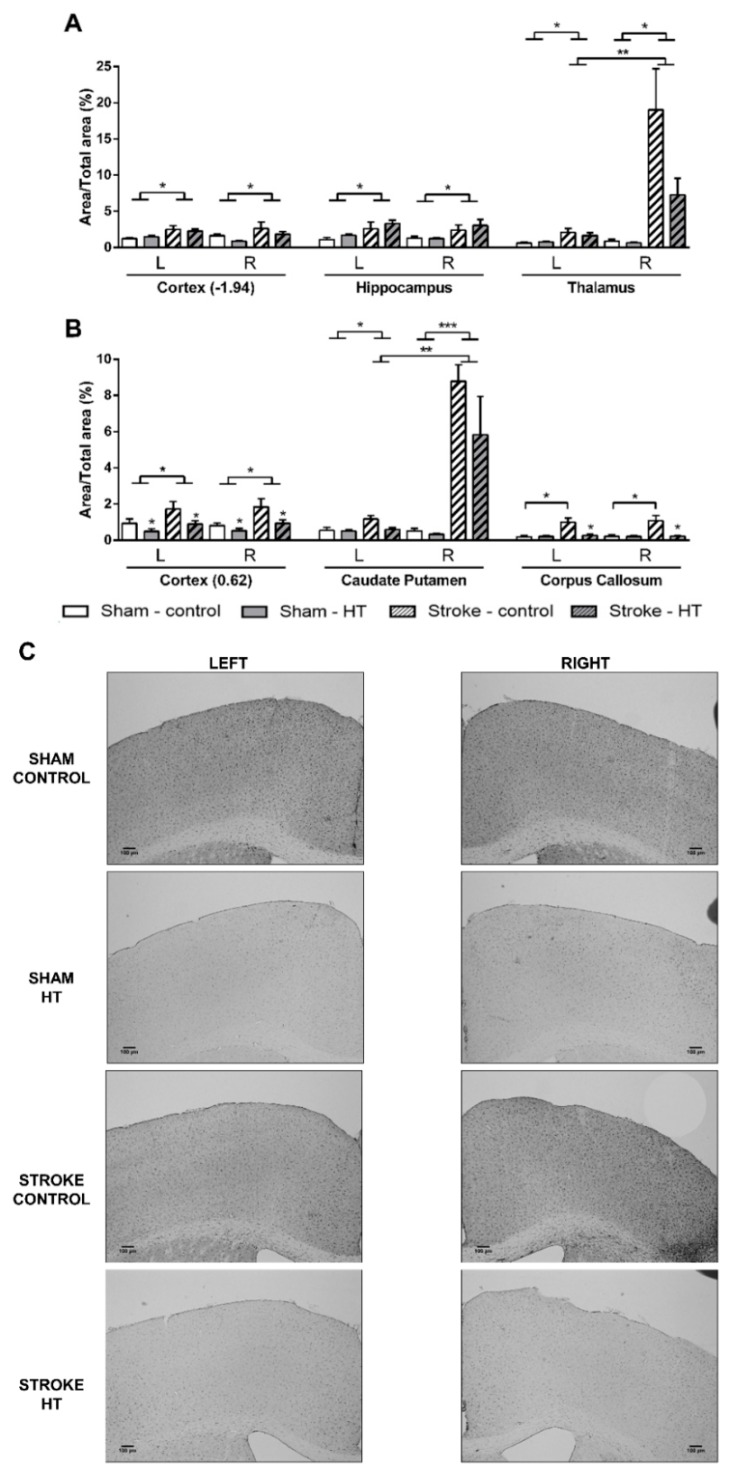
Immunohistochemical stainings for ionized calcium-binding adapter molecule-1 (IBA-1) in brains of HT and control-fed mice 35 days after surgery. All stroke mice showed a higher IBA1+-area than sham mice in (**A**) the cortex (bregma −1.94) (*p <* 0.046), hippocampus (*p <* 0.019), in both left (*p <* 0.002) and right (*p <* 0.002) thalamus, (**B**) cortex (bregma 0.62) (*p <* 0.027), and in both left (*p <* 0.037) and right (*p <* 0.001) caudate putamen. (**B**) Notably, in the corpus callosum, only in stroke-control mice a heightened IBA1+-area was found compared to sham-control mice (*p <* 0.011). Moreover, only in stroke mice in the right thalamus (*p <* 0.006) and in the right caudate putamen (*p <* 0.001) IBA1+-area was increased compared to their corresponding left hemispheric part. In the cortex at bregma 0.62, HT diet lowered IBA1+-area compared to control diet in both sham and stroke mice (*p <* 0.039). In the corpus callosum, IBA1+-area was decreased by HT diet only in stroke mice (*p <* 0.022). (**C**) Representative images of IBA-1 staining in cortex (bregma 0.62). Values represent mean ± SEM. * *p <* 0.05, ** *p <* 0.01, *** *p <* 0.001.

All stroke mice showed an higher IBA1+-area (Figure 14) than sham mice in the cortex at bregma −1.94 (F(1,21) = 4.5, *p <* 0.046), hippocampus (F(1,19) = 6.6, *p <* 0.019), in both left (F(1,23) = 10.9, *p <* 0.002) and right (F(1,23) = 11.6, *p <* 0.002) thalamus, cortex at bregma 0.62 (F(1,21) = 5.6, *p <* 0.027), and in both left (F(1,23) = 5.0, *p <* 0.037) and right (F(1,23) = 35.8, *p <* 0.001) caudate putamen. Notably, in the corpus callosum, only in stroke-control mice was a heightened IBA1+-area found compared to sham-control mice (F(1,13) = 8.9, *p <* 0.011). Moreover, only in stroke mice in the right thalamus (F(1,12) = 11.7, *p <* 0.006) and in the right caudate putamen (F(1,12) = 34.6, *p <* 0.001) IBA1+-area was increased compared to their corresponding left hemispheric part. In the cortex at bregma 0.62 HT diet lowered IBA1+-area compared to control diet in both sham and stroke mice (F(1,21) = 4.9, *p <* 0.039). In the corpus callosum, IBA1+-area was decreased by HT diet only in stroke mice (F(1,12) = 6.9, *p <* 0.022).

#### 3.4.3. GLUT-1 Staining

**Figure 15:**
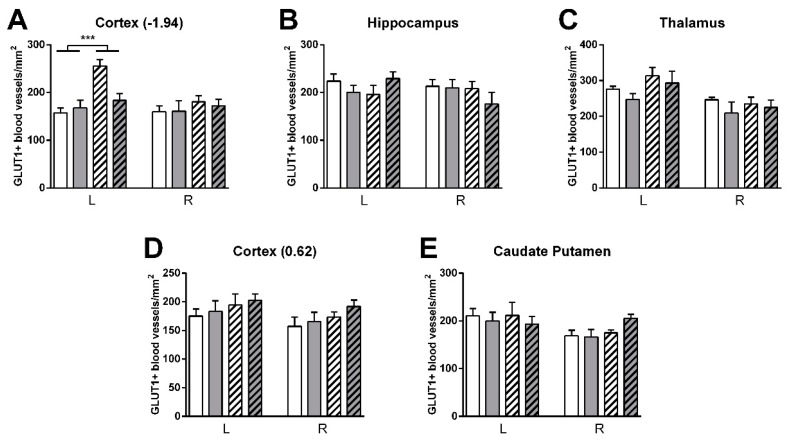
Immunohistochemical stainings for GLUT-1 in brains of HT and control fed mice 35 days after surgery. (**A**–**E**) Vascular density was increased in control stroke compared to control shams in the left cortex (bregma −1.94) (*p <* 0.001). HT stroke mice had a lower vascular density in the left cortex (bregma 0.62) than control stroke mice (*p <* 0.040, data not shown). Values represent mean ± SEM. *** *p <* 0.001.

**Figure 16:**
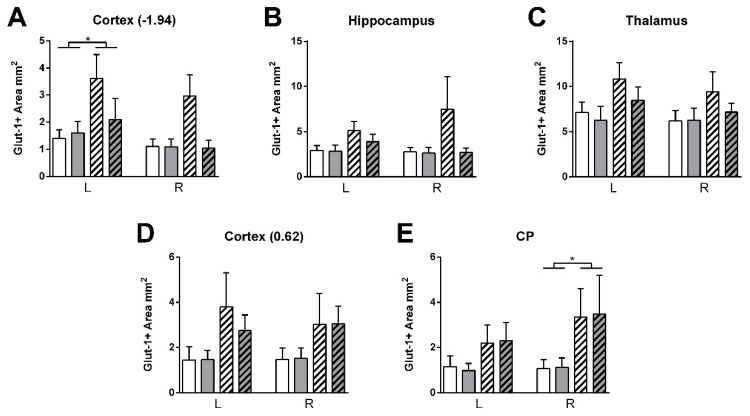
Immunohistochemical stainings for GLUT-1 in brains of HT and control fed mice 35 days after surgery. (**A**–**E**) GLUT-1+-area was increased in stroke mice compared to sham mice in left cortex (bregma −1.94: *p <* 0.046) and right caudate putamen (*p <* 0.043). Values represent mean ± SEM. * *p <* 0.05.

Vascular density was higher in stroke than shams in the left cortex (bregma −1.94) (F(1,23) = 17.3, *p <* 0.001) (Figure 15). HT stroke mice had a lower vascular density in the left cortex (bregma −1.94) than control stroke mice (F(1,23) = 4.8, *p <* 0.040, data not shown). Additionally, GLUT-1+-area was increased in stroke mice compared to sham mice in left cortex (bregma −1.94: F(1,23) = 4.5, *p <* 0.046) and right caudate putamen (F(1,22) = 4.6, *p <* 0.043) (Figure 16).

#### 3.4.4. Nitric oxide (NO) and Reactive Oxygen Species (ROS) Levels

**Figure 17:**
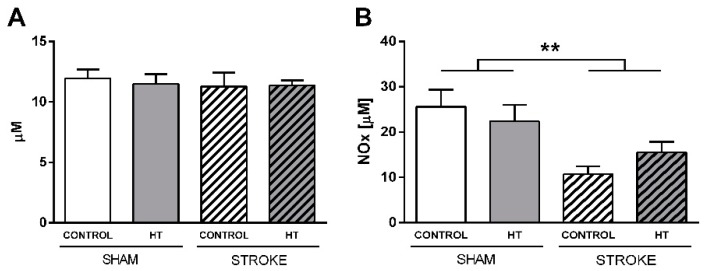
Nitric oxide (NO) and Reactive Oxygen Species (ROS) in serum samples obtained 35 days after surgery. (**A**) ROS level evaluated by analyzing Thiobarbituric acid reactive substances (TBARS) (**B**) NO production quantified by using an ozone chemiluminescence-based assay. A reduction of NO levels was detected in stroke animals (*p <* 0.002). Values represent mean ± SEM. ** *p <* 0.01.

NO and ROS levels were determined in serum samples obtained before sacrifice (Figure 17). NO production was quantified indirectly by the determination of nitrates/nitrites using an ozone chemiluminescence–based assay. A reduction in NO levels was detected in stroke animals with no evident diet effect (F(1,23) = 12.6, *p <* 0.002). ROS levels were also indirectly quantified by analyzing the level of lipid peroxidation. No changes were detected.

#### 3.4.5. Psd95, Bdnf, and GLUT-1 mRNA Expression

**Figure 18:**
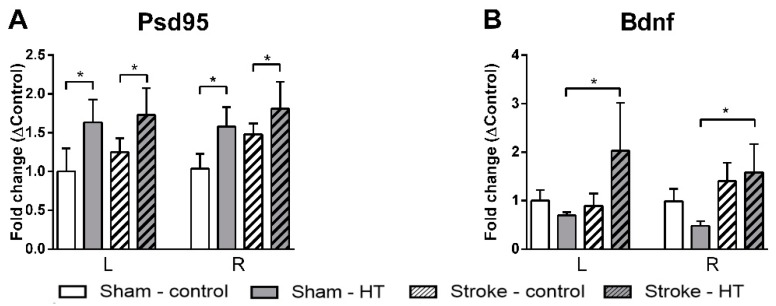
mRNA expression of (**A**) postsynaptic density protein 95 (Psd95), (**B**) brain derived neurotrophic factor (Bdnf) in frontal parts of the brain 35 days after surgery. (**A**) All HT-mice showed an up-regulation in Psd-95 expression without differences between hemispheres (*p <* 0.018). (**B**) Bdnf was higher expressed in stroke HT-mice (*p <* 0.018) than in sham-HT mice. Values represent mean ± SEM. * *p <* 0.05.

The expression of Bdnf, as a regulator of neurogenesis, and Psd-95, as postsynaptic marker, were determined by qPCR (Figure 18). All HT-mice showed an up-regulation in Psd-95 expression without differences between hemispheres (F(1,23) = 6.5, *p <* 0.018). In addition, Bdnf was higher expressed in stroke HT-mice (F(1,10) = 5.6, *p <* 0.040) than in sham HT-mice. The effect of HT in vascular integrity was evaluated by quantifying the mRNA levels of GLUT-1 as a capillary density marker. No changes were detected (data not shown).

## 4. Discussion

Although ischemic stroke is one of the main causes of death and disability worldwide, the only medical treatment for this disease is reperfusion by using recombinant tissue plasminogen activator or by endovascular thrombectomy with medical devices. However, the risk of hemorrhage and the narrow therapeutic window make it urgent to find other treatment options focused not only on reperfusion but on neuroprotection as well. In this sense, it has been previously described that olive oil and an olive leaf extract exert a neuroprotective effect in ischemic rats [12,43]. Oleuropein, another polyphenol from olive oil and a precursor of HT, has also been demonstrated to be neuroprotective in a mouse model of focal cerebral ischemia [44]. However, a comprehensive analysis of the effect of HT as a therapeutic approach in an in vivo stroke model is lacking. The HT concentration in EVOO is highly variable but can reach over 74.3 mg/kg [45]. It should be noted that these values do not take into account the HT available from oleuropein and oleuropein-aglycone. Therefore, the total exposure to HT may be estimated to be six times higher than the exposure to only free HT [46]. A daily intake of 50 mL of EVOO will mean an consumption of about 20 mg HT/day from this source [11]. Furthermore, this dose can be safely supplemented due to its very low toxicity [47]. In this context, the present study shows that an HT-enriched diet with a nutraceutical relevant dose of HT [25,26,27] could be used as a therapeutic approach in stroke recovery by improving (i) learning, short term memory, and grip strength; (ii) CBF and FC; and (iii) different parameters related to neurogenesis and neuroinflammation.

Motor functions, behavior, and cognition are severely affected after focal ischemia and decisive in the quality of life of stroke patients [48,49]. In our previous study, we described that a multicomponent diet, Fortasyn, improved grip strength in male mice [9]. In the current study, we show that HT improved grip strength on the trapeze, highlighting the potential restorative effect of this single dietary compound on motor network connections. Exploring the HT effects on behavior and motor skills, no diet effects were detected in the open field, DVC, nor the pole tests. Notably, in this study, DVC were used for the first time to study individual mouse locomotion via calculation of DVC metric measures (activity, walked distance, walked velocity, total turns, laterality index) during the day and night time before and after surgery. This novel approach helped to reveal decreased nighttime activity in stroke mice one week after surgery. Notably, during the nighttime of the second post-surgery week, only stroke mice showed a left turning preference. This latter result is in line with standard behavioral tests like the corner test used in preclinical stroke studies [48,50]. Short-term memory of mice was also evaluated with the novel object recognition test (ORT). Here, mice on HT diet visited the novel object more frequently than mice on the control diet. Similarly, in the PPI test, HT-fed mice showed a higher startle amplitude to the basal and final startle stimulus of 120 dB control mice. Altogether, the ORT and PPI results suggest that HT improves short-term memory and promotes non-associative learning processes such as habituation, being a promising approach to reduce stroke-associated cognitive deficits.

Moreover, with rsfMRI alterations in neuronal functional architecture, both in animal models and in humans, has been found after ischemic stroke [51]. In a human study, it was demonstrated that the alteration of sensorimotor function after a stroke correlated with a loss of interhemispheric connectivity between sensory-motor regions, and that this disruption normalized partially weeks after the infarction [52]. Thus, in patients with stroke, changes in neuronal activity are closely associated with functional recovery. The increase in rsfMRI activity in the supplementary motor cortex, the lateral premotor cortex and the superior parietal cortex in the first 14 days after infarction correlates with an improvement in motor function of the upper extremities during this period [53]. In the present study, we also investigated brain diffusivity with DTI as an imaging biomarker for white and grey matter (GM) integrity. Here, we revealed only impaired GM microstructure in the stroke mice on control diet measured by a decreased FA accompanied by an increased MD at seven days post-stroke in the right hippocampus and right motor cortex compared to their corresponding left counterpart. This effect was not visible in stroke mice on the HT diet, which is in line with our previous study in which a multicomponent diet improved functional and structural connectivity after stroke [9].

In our study, the HT diet improved functional and structural connectivity between several cerebral regions in the stroke animals. We suggest that these improvements in connectivity could be related to the increase in the grip strength of the HT-fed mice as well as with their higher habituation and improved short-term memory described above and on the up-regulation of different neurogenic markers that will be discussed later.

CBF alterations are also clinically associated with cognitive and motor dysfunction, especially after stroke [54]. In fact, we already described that a post-stroke diet intervention can improve CBF [9,55]. Other phenolic compounds such as resveratrol have shown to increase CBF in the frontal cortex of healthy humans after a task performance [56]. Moreover, in a rat ischemic model, resveratrol increased hippocampal CBF [57]. Our results show that an acute therapeutic approach with an HT diet was able to significantly increase CBF in the right hippocampus of all mice on HT diet, and to mitigate the decreased CBF in the left hippocampus stroke-control mice. Additionally, in the left cortex, the HT diet also increased CBF after stroke. The positive effect of HT on this parameter probably underlies the improvement in short-term memory and learning processes described above.

CBF is linked to a balanced production of NO. The particular effect of NO varies depending on the stage of evolution along the ischemic process and on the cellular source of NO [58,59]. Of the three NOS isoforms responsible for NO production, the activity of neuronal NOS and inducible NOS results are detrimental while endothelial NOS activation is related with neuroprotective effects. We have previously shown in stroke patients that an initial elevation of NO favors neurological recovery while a latter elevation predicts growth of the infarct volume [60]. In the present study, we have observed a significant decrease in serum NO concentration in stroke animals. This decrease has also been reported by us and by other research groups and may be attributed to the low profile of L-arginine, the NO precursor, in stroke patients [60,61,62].

A burst in ROS follows the stroke insult damaging cellular macromolecules and leading to cell death and tissue loss [63]. HT has been consistently described as an antioxidant compound in several models [64]. Therefore, we evaluated if the HT diet was able to modulate the oxidative level in serum samples obtained from mice after sacrifice. The fact that no changes were detected in any experimental group, not even between sham and stroke animals, seems to indicate that 35 days after surgery may be too late to detect changes in serum ROS levels. In fact, in a previous study with hypoxic mice we observed that ROS brain levels begin to normalize two hours after the insult [65]. As previously mentioned, further analysis with more animals should be carried out, both in serum and in brain samples, and at time-points closer to surgery to re-evaluate the temporal profile of NO production and the particular isoenzymes of NOS modulated by HT and to analyze the antioxidant capacity of an HT diet after stroke.

HT has shown its anti-inflammatory capacity in different models [66,67,68], although its particular effect on microglia-mediated neuroinflammation remained unexplored. Other phenolic compounds such as oleuropein have been shown to attenuate microglia activation [69]. The decreased level of IBA-1 immunoreactivity 35 days after stroke in the cortex and corpus callosum of mice on the HT diet corroborates that HT can also reduce the inflammatory environment after stroke. This effect is probably involved in the improved impairments of stroke mice on an HT diet and points to the interest of carrying out future experiments to deepen the investigation into the activity of HT on neuroinflammation.

Neurogenesis is an important process in stroke recovery and a number of therapeutic strategies to promote this process after ischemic events have been investigated with poor outcomes [70]. Stroke insult also involves synaptic degradation which dampens the activity of the CNS. Bdnf is a neurotrophin that regulates synaptic connections, synapse structure, neurotransmitter release and synaptic plasticity [71]. Moreover, Bdnf is required for the induction of neurogenesis and lack of this protein can lead to a lack of neurogenic response in a heterozygous knockout mice model [72]. Additionally, the postsynaptic protein Psd95 is also involved in the regulation of synaptic plasticity and synaptogenesis. Previous studies demonstrate that the administration of olive leaf or oil polyphenol extracts increases Bdnf levels in the olfactory lobes and hippocampus [73,74]. The synaptogenic potential of HT has been also reported in prenatally stressed rats in which HT prevented the stress-induced downregulation of Bdnf [75]. In accordance, in our study the expression of Psd95 was significantly induced in all HT-fed mice. Unfortunately, no diet effect was found on amount of DCX+ cells. However, the HT diet only induced Bdnf expression in stroke HT-mice, suggesting a difference in the response between Bdnf and DCX [76]. Although further analyses at the protein level are necessary, it is remarkable that HT, a single compound, exhibits these promising effects.

## 5. Conclusions

The data presented here indicate that a post-stroke intervention with a HT-enriched diet favor the recovery after ischemic stroke by ameliorating stroke-associated learning and motor impairments. This effect, probably linked to an increase in CBF, functional and structural connectivity, and to its anti-inflammatory and neurogenic potential, makes HT an interesting and safe compound to be further tested in ischemic stroke treatment.

## Supplementary Materials

The following are available online at https://www.mdpi.com/2072-6643/11/10/2430/s1, Table S1. Excluded mice per experiment. Table S2. Sequence of the PCR primers used in this study. (Used abbreviations: Bdnf, brain derived neurotrophic factor; GLUT-1, glucose transporter 1; Psd-95, postsynaptic density protein 95; HPRT, hypoxanthine guanine phosphoribosyl transferase; B2M, beta-2 microglobulin). Figure S1. A detailed explanation of the calculations on the aforementioned DVC metric measures. Figure S2. Individual locomotion via digital ventilated cage (DVC) metrics measures. (A) activity, (B) walked distance, (C,D) walked velocity, (E) total turns and (F,G) laterality index, during day- and nighttime before and after surgery. No diet effects were found on DVC metrics. Notably, several stroke effects were found: i.e., During nighttime, only stroke mice were less active after surgery over time (*p*< 0.026) comparing presurgery with postsurgery week 1. Only during nighttime, stroke mice (*p*< 0.028) showed a left turning preference (laterality) comparing presurgery with postsurgery week
