# Supplementary material for: Hydroxytyrosol, the Major Phenolic Compound of Olive Oil, as an Acute Therapeutic Strategy after Ischemic Stroke
Source: Nutrients. 2019 Oct 11;11(10):2430. doi: 10.3390/nu11102430 (PMC6836045; doi:10.3390/nu11102430)
Supplement: Supplementary file 1 [file nutrients-11-02430-s001.pdf]

Table S1. Excluded mice per experiment.

Test	Sham-Control	Sham-HT	Stroke-Control	Stroke-HT
Open Field	0	0	0	0
Grip test	0	0	0	0
Pole test	0	0	2	1
Rotarod	0	0	1	0
Prepulse inhibition	0	1	1	0
Morris water maze	0	0	0	0
Novel object recognition test	0	0	0	0
Digital ventilated cages	0	0	0	0
MRI—Arterial spin labelling	0	1	1	1
MRI—Diffusion tensor imaging	0	1	1	0
Resting state functional MRI	1	1	2	1
qPCR—PSD95 & GLUT-1	0	0	1	1
qPCR—BDNF	0	1	1	1
Immunohistochemistry—DCX	0	0	1	0
Immunohistochemistry—IBA-1 (0.62)	0	2	1	0
Immunohistochemistry—IBA-1 (−1.94)	1	1	0	1
Immunohistochemistry - GLUT-1 (0.62)	0	1	2	0
Immunohistochemistry - GLUT-1 (-1.94)	0	0	2	0
Serum NO	0	0	1	0
Serum Oxidative Stress Level	0	0	0	0

Table S2. Sequence of the PCR primers used in this study. (Used abbreviations: Bdnf, brain derived neurotrophic factor; GLUT-1, glucose transporter 1; Psd-95, postsynaptic density protein 95; HPRT, hypoxanthine guanine phosphoribosyl transferase; B2M, beta-2 microglobulin).

Gene	Direction	Sequence (5′ to 3′)
BDNF	Forward	CTTCCTGCATCTGTTGGGGA
	Reverse	TGGTGGAACATTGTGGCTTTG
GLUT1	Forward	GATCCCAGCAGCAAGAAGGT
	Reverse	TAGCCGAACTGCAGTGATCC
PSD95	Forward	TGGATCACAGGGTCGAGAAGA
	Reverse	TTGGCACGGTCTTTGGTAGG
HPRT	Forward	TGATTAGCGATGATGAACCAGGT
	Reverse	AGCAAGTCTTTCAGTCCTGTCC
B2M	Forward	GATGTCAGATATGTCCTTCAGCA
	Reverse	TCACATGTCTCGATCCCAGT

Angle:

$$\alpha = acos\frac{|X_n - X_{n-1}|}{DM_n}$$

where X is x position and DM is direction of motion.

Relative Turn Angle: Change in heading between two consecutive samples

$$RTA_n = HE_n - HE_{n-1}$$

Relative Angular Velocity (synonym, Turn Bias): Change in relative turn angle over time delta

$$RAV_n = \frac{RTA_n}{t_n - t_{n-1}}$$

Laterality Index: Tendency to turn right vs left

$$LI = \frac{\|RTA_R\|}{\|RTA_R\| + \|RTA_L\|}$$

where RTA_R is a right turn angle $\in [30, 90]$, RTA_L is a left turn angle $\in [-30, -90]$, and $LI \in [0, 1]$.

Figure S1. A detailed explanation of the calculations on the aforementioned DVC (Digital Ventilated Cages) metric measures.

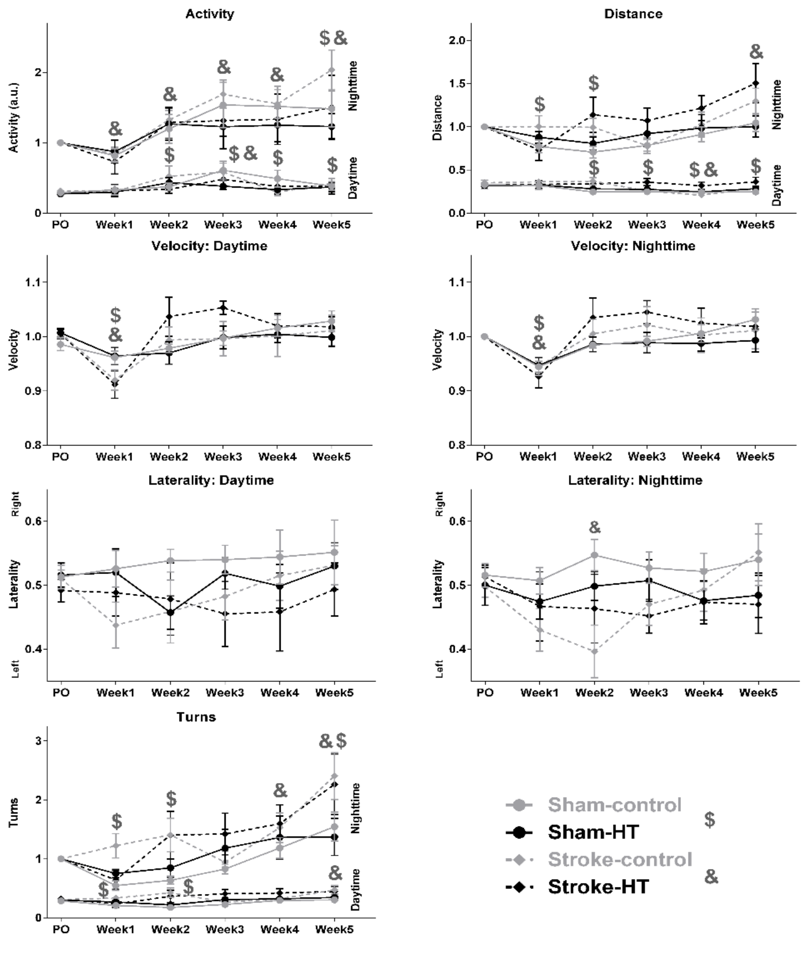

Figure S2. Individual locomotion via digital ventilated cage (DVC) metrics measures. (**A**) activity, (**B**) walked distance, (**C**,**D**) walked velocity, (**E**) total turns and (**F**,**G**) laterality index, during day- and nighttime before and after surgery. No diet effects were found on DVC metrics. Notably, several stroke effects were found: i.e., During nighttime, only stroke mice were less active after surgery over time ($p < 0.026$) comparing presurgery with postsurgery week 1. Only during nighttime, stroke mice ($p < 0.028$) showed a left turning preference (laterality) comparing presurgery with postsurgery week 2.
